# Late Maternal Folate Supplementation Rescues from Methyl Donor Deficiency-Associated Brain Defects by Restoring Let-7 and miR-34 Pathways

**DOI:** 10.1007/s12035-016-0035-8

**Published:** 2016-08-17

**Authors:** Andréa Geoffroy, Racha Kerek, Grégory Pourié, Déborah Helle, Jean-Louis Guéant, Jean-Luc Daval, Carine Bossenmeyer-Pourié

**Affiliations:** 1 Inserm U954, 9 Avenue de la Forêt de Haye, 54500 Vandoeuvre-lès-Nancy, France; 2 Faculté de Médecine de Nancy, Université de Lorraine, 54500 Vandoeuvre-lès-Nancy, France; 3 IRCCS, Oasi Maria S.S., Institute for Research on Mental Retardation and Brain Aging, 94018 Troina, EN Italy

**Keywords:** Brain development, Folate, Vitamin B12, microRNAs, Epigenetics, Belated folate supplementation

## Abstract

The micronutrients folate and vitamin B12 are essential for the proper development of the central nervous system, and their deficiency during pregnancy has been associated with a wide range of disorders. They act as methyl donors in the one-carbon metabolism which critically influences epigenetic mechanisms. In order to depict further underlying mechanisms, we investigated the role of let-7 and miR-34, two microRNAs regulated by methylation, on a rat model of maternal deficiency. In several countries, public health policies recommend periconceptional supplementation with folic acid. However, the question about the duration and periodicity of supplementation remains. We therefore tested maternal supply (3 mg/kg/day) during the last third of gestation from embryonic days (E) 13 to 20. Methyl donor deficiency-related developmental disorders at E20, including cerebellar and interhemispheric suture defects and atrophy of selective cerebral layers, were associated with increased brain expression (by 2.5-fold) of let-7a and miR-34a, with subsequent downregulation of their regulatory targets such as Trim71 and Notch signaling partners, respectively. These processes could be reversed by siRNA strategy in differentiating neuroprogenitors lacking folate, with improvement of their morphological characteristics. While folic acid supplementation helped restoring the levels of let-7a and miR-34a and their downstream targets, it led to a reduction of structural and functional defects taking place during the perinatal period. Our data outline the potential role of let-7 and miR-34 and their related signaling pathways in the developmental defects following gestational methyl donor deficiency and support the likely usefulness of late folate supplementation in at risk women.

**Electronic supplementary material:**

The online version of this article (doi:10.1007/s12035-016-0035-8) contains supplementary material, which is available to authorized users.

## Introduction

Embryogenesis implies a series of programming processes involving accurate time-controlled gene activation/silencing expressions. Numerous studies have shown that epigenetic regulations such as DNA methylation and histone modification play a critical role on genomic programming during embryogenesis and fetal development by regulating cell proliferation and differentiation [1, 2]. Epigenetic mechanisms also serve as important interfaces between genes and environmental cues [3, 4]. In this respect, epigenetic variability appears to be susceptible to modulation by exogenous factors such as nutritional components, and early exposure to inappropriate maternal diet may lead to developmental disorders and increased risk for certain diseases later in life [5, 6].

Among the “epigenetic dietary compounds” identified, the methyl donors folate (vitamin B9) and vitamin B12 are major actors in the one-carbon metabolism that plays a key role in epigenetic regulations, including during development [7, 8]. Indeed, both vitamins are essential for the transfer of one-carbon units for synthesis of S-adenosylmethionine (SAM), the universal methyl donor for biological methylations [9], and thus, their deficiency results in decreased methylation capacities [9, 10]. Moreover, B vitamins participate to the regulation of homocysteine concentration. Folate and vitamin B12 are essential for the development of the central nervous system, and a reduced status in these compounds has been associated with various developmental effects, ranging from brain growth retardation to major congenital disorders such as neural tube defects (NTDs), including spina bifida and anencephaly [11–14]. The protective effect of folate on the occurrence of NTDs has been clearly demonstrated, leading to national public health policies recommending periconceptional supplementation with folic acid [15–18]. Owing to the role of folate in the methylation pathway, it is accepted that disturbed methylation may significantly account for the relation between NTDs and folate [19, 20], reinforcing the epigenetic hypothesis [21, 22].

MicroRNAs (miRNAs) are small noncoding RNAs that regulate posttranscriptional gene expression. The presence of specific miRNAs is enriched in the central nervous system where they influence cell cycling, cell differentiation, and apoptosis during development [23–26]. It has been reported that folate deficiency and DNA hypomethylation can lead to misexpression of miRNAs which in turn may substantially affect neural development [27]. Among the subset of miRNAs known to be regulated by methylation [28], let-7 (*lethal 7*) and miR-34 are believed to exert a requisite role at various steps of cerebral development, while they would influence the occurrence of NTDs [27, 29].

We recently showed that methyl donor deficiency (folate and B12) during the embryofetal period in the rat causes overexpression of miR-124, resulting in the loss of Stat3 signaling and in a substantial impairment of brain development [14]. In order to identify further mechanisms underlying the effect of maternal B-vitamin status on neural tube and brain development, in line with potential epigenetic dysregulations, we investigated the participation of let-7 and miR-34 as well as their related pathways in the consequences of methyl donor deficiency both in vivo on a validated rat model of maternal deficiency [30, 31] and in vitro in hippocampal progenitors [32]. In addition, since folate supplementation is classically prescribed to women until the end of the first trimester of pregnancy in several countries, we aimed to test whether a later vitamin supply (i.e., during the last trimester, corresponding to the third week of gestation in the rat) could exert beneficial effects on progeny brain outcome.

## Materials and Methods

### Animals and Tissue Collection

In vivo experiments were performed on a validated animal model of methyl donor deficiency [30, 31]. They were conducted in compliance with the international guidelines for the care and use of laboratory animals and were approved by the local University Research Ethics Board. Wistar rats (Charles River, l’Arbresle, France) were maintained under standard laboratory conditions, on a 12-h light/dark cycle, with food and water available ad libitum. One month before mating, adult females were fed either a standard diet (Maintenance diet M20, Scientific Animal Food and Engineering, Villemoisson-sur-Orge, France) or a methyl donor-deficient (MDD) low-choline diet (119 vs 1780 mg/kg) lacking folate and vitamin B12 (Special Diet Service, Saint-Gratien, France). Methionine content (∼0.4 %) was similar in both diets. In the supplementation protocol, folic acid (the synthetic form of folate, Sigma-Aldrich, Saint-Quentin Fallavier, France) diluted in condensed milk was given per OS at the dose of 3 mg/kg per day in a final volume of 1 mL to dams from embryonic days (E) 13 to 20. Matched control dams received the same volume of vehicle (i.e., 1 mL condensed milk) over the same period. Whatever the maternal diet, fetuses were collected at E9, E13, E16, or E20. They were euthanized by excess isoflurane, and then weighed and evaluated morphologically with the aid of a BX51WI microscope (Olympus, Rungis, France) coupled to a ProgRes MF cool camera (Jenoptik, Jena, Germany). For biochemical analyses, the brains were rapidly harvested and the midbrain was dissected before freezing in liquid nitrogen and stored at −80 °C. For immunochemistry, brains were immediately fixed in 4 %-paraformaldehyde (24–48 h) at 4 °C, dehydrated, and included in paraffin. Microtome-generated 12-μm sagittal brain sections were then mounted onto glass slides and stored at ambient temperature. In addition, blood samples were collected from dams for subsequent analyses.

### Measurement of Maternal Plasma Concentrations of Homocysteine, Vitamin B12 and Folate and Offspring Tissue Concentrations of SAM and SAH

Homocysteine concentrations were measured by HPLC (Waters, St. Quentin, France) coupled with mass spectrometry (Api 4000 Qtrap; Applied Biosystems, Courtaboeuf, France) [33]. Vitamin B12 and folate concentrations were measured by radio-dilution isotope assay (simulTRAC-SNB; ICN Pharmaceuticals, Versailles, France) [34]. S-adenosylmethionine (SAM) and S-adenosylhomocysteine (SAH) concentrations were measured in tissue homogenates using high-performance liquid chromatography adapted from Delabar et al. [35]. Proteins were precipitated with 0.2N HClO4 before injection on the column (Lichrospher, 100 RP-C18, 5 μm, 250 × 4 mm ID). The mobile phase, consisting of 50 mmol/L sodium phosphate (pH 3.2), 10 mmol/L heptane sulfonate, and acetonitrile (10–20 % from 0 to 20 min), was applied at 0.75 mL/min flow rate. Amounts of SAM and SAH were quantified using a UV detector set at 254 nm.

### DNA Methylation

Global DNA methylation was quantified in brain tissues at E20 by using the Methylamp™ Global DNA Methylation Quantification Kit (Epigentek, Euromedex, Mundolsheim, France), as previously described [36]. According to the manufacturer’s instructions, the methylated fraction of DNA can be recognized by a 5-methylcytosine antibody and quantified through an ELISA-like reaction. The amount of methylated DNA is proportional to the OD intensity.

### Histopathological Analyses

For basic histopathological investigations, brain sections were stained with the DNA fluorochrome 4,6-diamidino-2-phenylindole (Dapi, Sigma-Aldrich) for the measurement of thickness of specific brain layers.

For skeleton analysis, the procedure adapted from Wallin et al. [37] involves complete skinning of fresh eviscerated rat embryos following a 24-h immersion in 95 % ethanol. Skeletons were stained for 48 h with 1 % Alcian blue dye, diluted in an ethanol/acetic acid mix, specific for cartilage staining. Skeletons were then macerated in 2 % KOH until bones are visible and were stained for 24 h with 0.12 % Alizarin red dye diluted in KOH, specific for bone staining. Specimen were finally cleared and hardened in glycerol/ethanol baths and stored in 87 % glycerol. Detailed observations and measurements were performed by means of the Cell® software (Olympus).

### Cell Cultures

H19-7/IGF-IR cell line (ATCC# CRL-2526) was conditionally immortalized from embryonic day 17 rat hippocampi with a temperature-sensitive simian virus 40 (SV40) large tumor antigen [38]. Cells were seeded at a density of 3 × 10^5^ cells per well in 24-well plates (Falcon, Dutscher, Brumath, France) precoated with poly-L-lysine in high-glucose Dulbecco’s modified Eagle’s medium (DMEM) supplemented with 10 % fetal calf serum, 50 U/mL penicillin, 50 U/mL streptomycin, 2 mM glutamine, and 1 mM sodium pyruvate, as previously described [32, 39]. For homogeneity, the same lot of serum was used throughout all experiments. Cells were allowed to proliferate at the temperature of 33 °C in 95 % air/5 % CO_2_. Selection was maintained with 200 μg/mL G418. Because standard DMEM does not contain vitamin B12, methyl donor deficiency was induced by using DMEM lacking vitamin B9 (Invitrogen, Cergy-Pontoise, France). After 24 h, cells were washed and shifted to the non-permissive temperature (39 °C) for induction of their differentiation in high-glucose DMEM (with or without B9) containing 1 % fetal calf serum, N2 supplement (Invitrogen), 0.11 mg/mL sodium pyruvate, and 2 mM glutamine.

### Immunohistochemistry

In addition to brain sections used for immunohistological analysis, cells cultured on poly-L-lysine-precoated glass coverslips were washed, fixed with 4 % paraformaldehyde, and permeabilized with 0.1 % Triton X-100 (Sigma-Aldrich), as reported by Akchiche et al. [32] and Kerek et al. [14]. Nonspecific binding sites were blocked in phosphate-buffered saline containing 1 % bovine serum albumin (BSA), and incubation was performed overnight with an antibody against one of the following proteins: Trim71 (goat polyclonal, 1/200, USBiological, Euromedex, Souffelweyersheim, France), Dll1 (sheep polyclonal, 1/200, USBiological), Notch1 (Rabbit monoclonal, 1/200, Cell Signaling Technology), Hes1 (rabbit monoclonal, 1/200, Cell Signaling Technology), Mash1 (rabbit polyclonal, 1/200, Abcam), actin (goat polyclonal, 1/200, Santa Cruz Biotechnology, Clinisciences, Nanterre, France), and NF68 (rabbit polyclonal, 1/300, USBiological). After a washing step, immunoreactivity was assessed by incubation in the presence of an appropriate secondary anti-IgG antibody conjugated to AlexaFluor for 1 h at 25 °C (1/1000, Life Technologies, Saint-Aubin, France). Control experiments were conducted by omitting the primary antibody. Immunofluorescence visualization, image acquisition (×20 and ×60 magnification), and unbiased cell counts in randomly selected fields were performed with a BX51WI microscope (Olympus) coupled to a ProgRes MF cool camera (Jenoptik) or a confocal microscope (Nikon Instruments, Champigny sur Marne, France) and analyzed by Cell® software.

### Western Blotting

Following protein extraction with RIPA buffer, Western blot analyses were performed using standard procedure with chemiluminescence using ECL system (Bio-Rad, Marnes-la-Coquette, France), as previously detailed [39]. Antibodies against the following proteins were used: Trim71 (goat polyclonal, 1/1000, LSBio, Clinisciences), Dll1 (sheep polyclonal, 1/1000, USBiological), Notch1 (Rabbit monoclonal, 1/1000, Cell Signaling Technology), Hes1 (rabbit monoclonal, 1/1000, Cell Signaling Technology), and Mash1 (rabbit polyclonal, 1/1000, Abcam, Paris, France).

Glyceraldehyde-3-phosphate dehydrogenase (GAPDH, chicken monoclonal, 1/1000, Millipore, Fontenay-sous-Bois, France) was used as an internal standard. Polyvinylidenedifluoride membranes were incubated for 1 h at room temperature with the corresponding horseradish peroxidase-conjugated preadsorbed secondary antibody (1/2000, Santa Cruz).

### RNA Extraction

Total RNA was extracted from 0.5 mg of embryonic midbrain tissues and from H19-7 cells (10^2^ to 10^7^ cells) and using the mirVana®miRNA Isolation kit (Applied Biosystems, Foster City, CA, USA) following the manufacturer’s instructions and as previously described by Kerek et al. [14]. miRNAs were isolated using a two-step procedure. In the first step, samples were disrupted in a denaturing lysis buffer and then subjected to acid-phenol/chloroform extraction. The second step consisted on purification over glass-fiber filter that immobilizes the RNA which was later eluted using RNase-free water. According to the manufacturer’s instructions, no enrichment procedure is needed while isolating miRNA for expression profiling using miRNA arrays. The concentration and purity of RNA were determined at 260/280 nm by using a nanodrop spectrophotometer (Multiskan GO).

### Analysis of Let-7a and miR-34a Expression

#### TaqMan RT-qPCR

Two-step real-time PCR was used to analyze the expression of microRNAs. In the first step, total RNA was reverse transcribed using miRNA-specific RT primers (rno-let-7, rno-miR-34, and U6SnoRNA) and a TaqMan® MicroRNA Reverse Transcription Kit (Applied Biosystems, Villebon-sur-Yvette, France). miRNA expression was analyzed using TaqMan microRNA assays (Applied Biosystems), according to the instructions of the manufacturer. The RT reaction was performed in 15 μL volume, containing 1 μg RNA sample, 3 μL primer, and master mix adjusted to 15 μL/reaction. Products of RT reaction (1.33 μL) were used in a real-time PCR reaction, which also included 10 μL of the TaqMan Universal Master Mix II, and 1 μL TaqMan miRNA assay containing the sequence-specific primers of either the target miRNA (let-7: UGAGGUAGUAGGUUGUAUAGUU, miR-34: UGGCAGUGUCUUAGCUGGUUGU) or the U6SnoRNA (CACGAATTTGCGTGTCATCCTT) used as an endogenous control for normalization. Real-time PCR was carried out by means of a Step One Plus Real-Time PCR System (Applied Biosystems). Incubations were performed in a 96-well plate at 95 °C for 10 min for enzyme activation, followed by 40 cycles of PCR: denaturation (95 °C for 15 s) and annealing/extending (60 °C for 2 min). Data analysis was performed with the software provided by the manufacturer (Step One Plus).

#### In Situ Hybridization

The in situ detection of let-7a and miR-34a was performed on paraffin-embedded sections from normal and methyl donor-deficient brain tissues by locked nucleic acid (LNA)-oligo in situ hybridization, as previously described by Kloosterman et al. [40]. Briefly, the slides were deparaffinized in xylene, rehydrated in decreasing concentrations of ethanol, and treated with proteinase-k for nucleic acid release. Slides were then redehydrated and prehybridized in hybridization buffer with 0.5 nm specific probe (LNA-modified and digoxygenin (DIG)-labeled oligonucleotide, Exiqon, Copenhagen, Denmark) complementary to let-7a (AACTATACAACCTACTACCTCA ) or miR-34a (ACAACCAGCTAAGACACTGCCA). Sections were then washed in saline sodium citrate buffer, followed by blocking in Denhardt solution 1× in a humidified chamber. Slides were then incubated with anti-DIG antibody (1/500, Roche Applied Science) for 1 h at room temperature, washed in PBS-T, and then immunoreactivity was assessed in the presence of a matching secondary antibody conjugated to AlexaFluor (1/2000, Molecular Probes) for 1 h at room temperature. Positive controls (snoRNA U6B, Exiqon: CACGAATTTGCGTGTCATCCTT) were used for each hybridization experiment.

### Small Interfering RNA (siRNA) and Cell Transfection

Let-7a and miR-34a expression in cell cultures was silenced using small interfering RNA. The siRNA oligonucleotide duplexes were purchased from Ambion (Applied Biosystems) for targeting the rat let-7a (hsa-let-7a-5p) or miR-34a (hsa-miR-34a-5p) in H19-7 cells. The siRNA sequence is (sense strand indicated): 5′-UGAGGUAGUAGGUUGUAUAGUU-3′ for let-7a, 5′-UGGCAGUGUCUUAGCUGGUUGU-3′ for miR-34a, and mirVana™ miRNA Inhibitor Negative Control #1 was used as control for evaluation of the effect of the experimental miRNA inhibition. siRNA duplexes (25 nM final concentration for the first transfection then 10 nM for the second day retransfection) were transfected with lipofectamine RNAiMax (Invitrogen). Three days post-transfection, cells were harvested for RNA or protein extraction.

### MicroRNA Targets RT^2^ Profiler PCR Arrays

Rat microRNA Targets RT^2^ Profiler PCR Arrays (Qiagen, Courtaboeuf, France) in a 96-well plate format were used to monitor the expression of the most relevant experimentally documented or bioinformatically predicted gene targets for let-7a and miR-34a. Biological samples were obtained from the brains of two to three E20 fetuses and were tested in duplicate in separate plates. Total RNA was purified using the RNeasyPlus Mini Kit (Qiagen). Following manufacturer’s instructions, total RNA was reverse transcribed using RT^2^ First Strand kit. Reaction was performed in the iCylcler instrument (Bio-Rad). The reaction includes a gDNA elimination step. The RT reaction was performed in a 20-μL volume containing 10 μL of gDNA-free RNA (500 ng RNA, 2 μL gDNA elimination buffer, and RNase-free water) and 10 μL of RT mix (4 μL RT buffer, 1 μL primer and external control, 2 μL RT enzyme mix, and 3 μL RNase-free water). Products of RT reaction were mixed with 91 μL of RNase-free water for a final template volume of 111 μL. For PCR reaction, 1350 μL RT^2^ SYBR Green mastermix and 1248 μL RNase-free water were added. Twenty-five microliters of PCR components were added to each well of the 96-well plate. Real-time PCR was carried out on a Step One Plus apparatus (Applied Biosystems). Plates were incubated at 95 °C for 10 min for enzyme activation, followed by 40 cycles of PCR: denaturation (95 °C for 15 s) and annealing/extending (60 °C for 1 min). Data analysis of the transcription of 84 separate genes was performed with the software provided by the manufacturer (Step One Plus).

### Behavioral Evaluation

As a global functional approach, the “homing test” [41] was performed in the offspring between postnatal days 5 and 16 in order to evaluate basic learning performances and locomotor capacities in the different experimental groups. For this purpose, the maternal diets were as described above and maintained after delivery until the last testing day.

The abilities of rat pups to successfully return to their home-cage by using environmental sensitive information were recorded in a T-maze, as previously described [42]. At each time point studied, rats performed one trial test consisting in moving freely in the T-maze area (30 cm in length for each arm, walls of 10 cm high, and a corridor of 5 cm wide). The home-cage (without the dam) was positioned at the end of one arm and a clean cage of the same size was positioned at the end of the opposite arm. The corridor of the maze was carefully washed between each animal. The test was considered successful when the pup returned directly to his home-cage without visiting the arm containing the clean cage and without returning to the starting arm. The time spent in the home area was recorded. For homogeneity, tests were always performed between 8:00 and 11:00 a.m.

### Statistical Analysis

Data were analyzed with Statview 5 software for Windows (SAS Institute, Berkley, CA, USA). They were compared by using one-way analysis of variance (ANOVA) with Fisher’s test. *P* value <0.05 was considered to indicate significance.

## Results

### Gestational Methyl Donor Deficiency Affects the One-Carbon Metabolism and Methylation Capacities that Are Improved by Folic Acid Supplementation

As expected, plasma levels of folate and vitamin B12 were dramatically reduced in gestating females exposed to the deficient diet. Concomitantly, homocysteinemia was significantly augmented (Table 1). Folic acid supplementation restored folate concentration and significantly reduced hyperhomocysteinemia in the deficient group. In brain tissue homogenates from E20 fetuses, SAM concentration remained unaffected by the maternal dietary conditions, whereas SAH concentration was enhanced by exposure to the deficient diet, in good accordance with previous studies [30, 43]. As a consequence, the SAM/SAH ratio, an index of methylation capacities, was reduced in deficient fetuses (Table 2). When the dams received folic acid supplementation, SAM/SAH ratio was significantly increased in the brains of deficient fetuses as compared to those exposed solely to methyl donor deficiency. Consistently, global DNA methylation was reduced by ∼50 % in the fetal brain under low methyl donor conditions, but was normalized following folic acid supplementation (Table 2).

**Table 1:** Effects of the dietary regimen on plasma concentrations of folate, vitamineB12, and resulting homocysteinemia in gestating females

	Control-vehicle	MDD-vehicle	Control + B9	MDD + B9
Plasma folate (nmol/L)	27.9 ± 8.3	4.6 ± 3.3**	37.1 ± 7.3*	27.8 ± 12.9°
Plasma vitamine B12 (pmol/L)	470.0 ± 137.6	208.3 ± 41.9**	400.8 ± 132.4	207.9 ± 44.1**
Plasma homocysteine (μmol/L)	4.2 ± 1.5	19.6 ± 5.4**	4.1 ± 0.8	9.9 ± 1.3**/°°

Blood samples were obtained at the time of collection of fetuses. Data are means ± SD and were obtained from 5 ≤ *n* ≤ 21 individuals. Statistically significant differences: **P < 0.01, with respective control; °*P <* 0.05 and °°*P <* 0.01, between MDD-vehicle and MDD + B9

*MDD* methyl donor deficient diet

**Table 2:** Effects of the maternal dietary regimen on SAM and SAH brain concentrations and on DNA global methylation in E20 fetuses

	Control-vehicle	MDD-vehicle	Control + B9	MDD + B9
Brain SAM (nmol/g tissue)	67.42 ± 2.94	65.72 ± 0.83	68.84 ± 0.85	69.34 ± 2.92
Brain SAH (nmol/g tissue)	2.51 ± 0.29	3.31 ± 0.53**	3.12 ± 0.16	3.14 ± 0.59
SAM/SAH ratio	25.45 ± 2.29	17.83 ± 2.11**	21.03 ± 0.88	22.59 ± 3.73°°
DNA methylation (%)	28.59 ± 5.74	13.86 ± 2.63**	27.91 ± 4.80	27.82 ± 7.78°°

Data are means ± SD and were obtained from 6 individuals. Statistically significant differences: *P < 0.05 and **P < 0.01, with respective control; °*P <* 0.05 and °°*P <* 0.01, between MDD-vehicle and MDD + B9

*MDD* methyl donor deficient diet, *SAM* S-adenosylmethionine, *SAH* S-adenosylhomocysteine

### Methyl Donor Deficiency Is Associated With Various Developmental Abnormalities and Defective Closure of the Posterior and Cephalic Parts of the Neural Tube: Beneficial Effects of Folic Acid Supplementation

Nutritional methyl donor deficiency starting 1 month prior to mating affected female ability to conceive. Globally, 45.7 % gave birth to pups (vs 84 % in controls). The number of live fetuses per litter was consistently reduced (6.7 vs 11.2). In addition to spontaneous abortions, maternal pup-killing behavior and cannibalism was more frequently observed in deficient dams, as previously documented for thiamine deficiency [44].

**Fig. 1:**
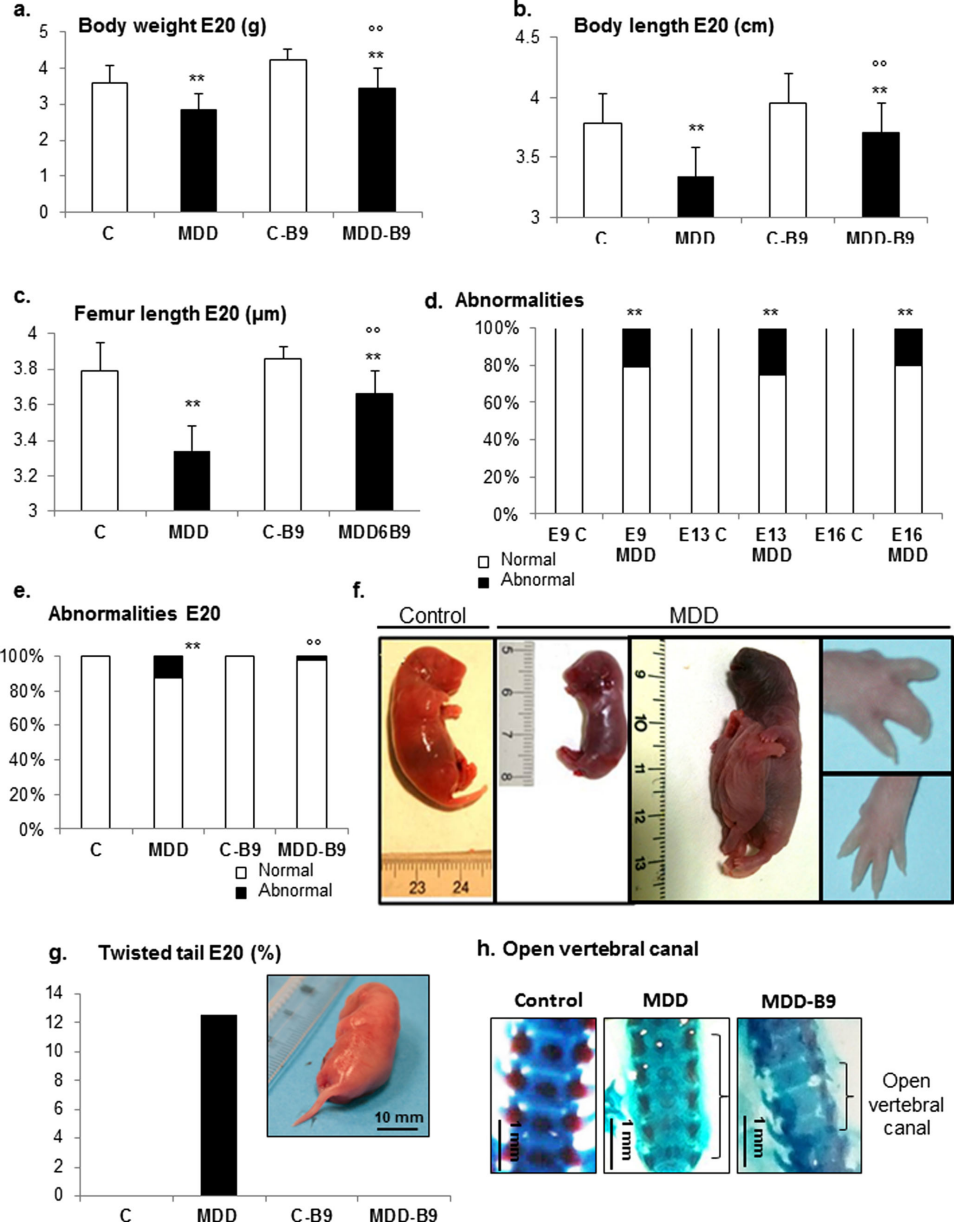
Effects of methyl donor deficiency and folic acid supplementation on rat fetus morphometric properties and developmental abnormalities. **a**–**c** General morphometric measurements in control (*C*), methyl donor deficient (*MDD*), supplemented control (*C-B9*), and supplemented deficient (*MDD-B9*) fetuses at embryonic day (*E*) 20 (10 ≤ *n* ≤ 81). Data are reported as means ± standard deviation. Statistically significant differences between control and MDD rats, **P < 0.01, and between MDD and supplemented MDD, °°*P* < 0.01. **d**, **e** Occurrence of developmental abnormalities in control and MDD fetuses at E9, E13, and E16 stages (15 ≤ *n* ≤ 38) and following folic acid supplementation at E20 (37 ≤ *n* ≤ 64). Statistically significant differences between control and MDD rats, **P < 0.01, and between MDD and supplemented MDD, °°*P* < 0.01. **f** Illustrations of developmental abnormalities occurring in methyl donor-deficient rats: growth retardation, conjoined fetuses, and digit malformations. **g** Prevalence of “twisted tail” in controls, MMD, and supplemented rats. **h** Photographs of the vertebral canal (Alcian blue/Alizarin red staining) in the various experimental groups (*square brackets* delineate open canal)

As previously documented in the same animal model [14], gestational deficiency was associated with global intrauterine growth retardation, as reflected by a significant reduction of body weight, body length, and femur length at the stage of E20 (Fig. 1a–c). This could be corrected at least partly by folic acid supplementation during the last third week of gestation. Morphologic abnormalities were detected in ∼20 % of deficient embryos at E9, E13, and E16, but not, or very exceptionally, in controls (Fig. 1d). They were still present at E20, but at a somewhat lower rate (Fig. 1e), presumably due to spontaneous abortions of the most affected fetuses. In addition to marked growth retardation, these abnormalities include syndactyly and related malformations such as atrophied digits, conjoined fetuses, as well as various signs indicative of spina bifida, such as “twisted tail” (12 %) and open vertebral canal (Fig. 1f–h), in addition to delayed ossification and fused vertebrae in the lumbosacral region. Following folic acid supplementation, the occurrence of abnormalities was consistently reduced (Fig. 1e). Whereas twisted tail was not detected in supplemented E20 fetuses, opening of the vertebral canal was still present, but to a smaller extent, as illustrated on Fig. 1h.

**Fig. 2:**
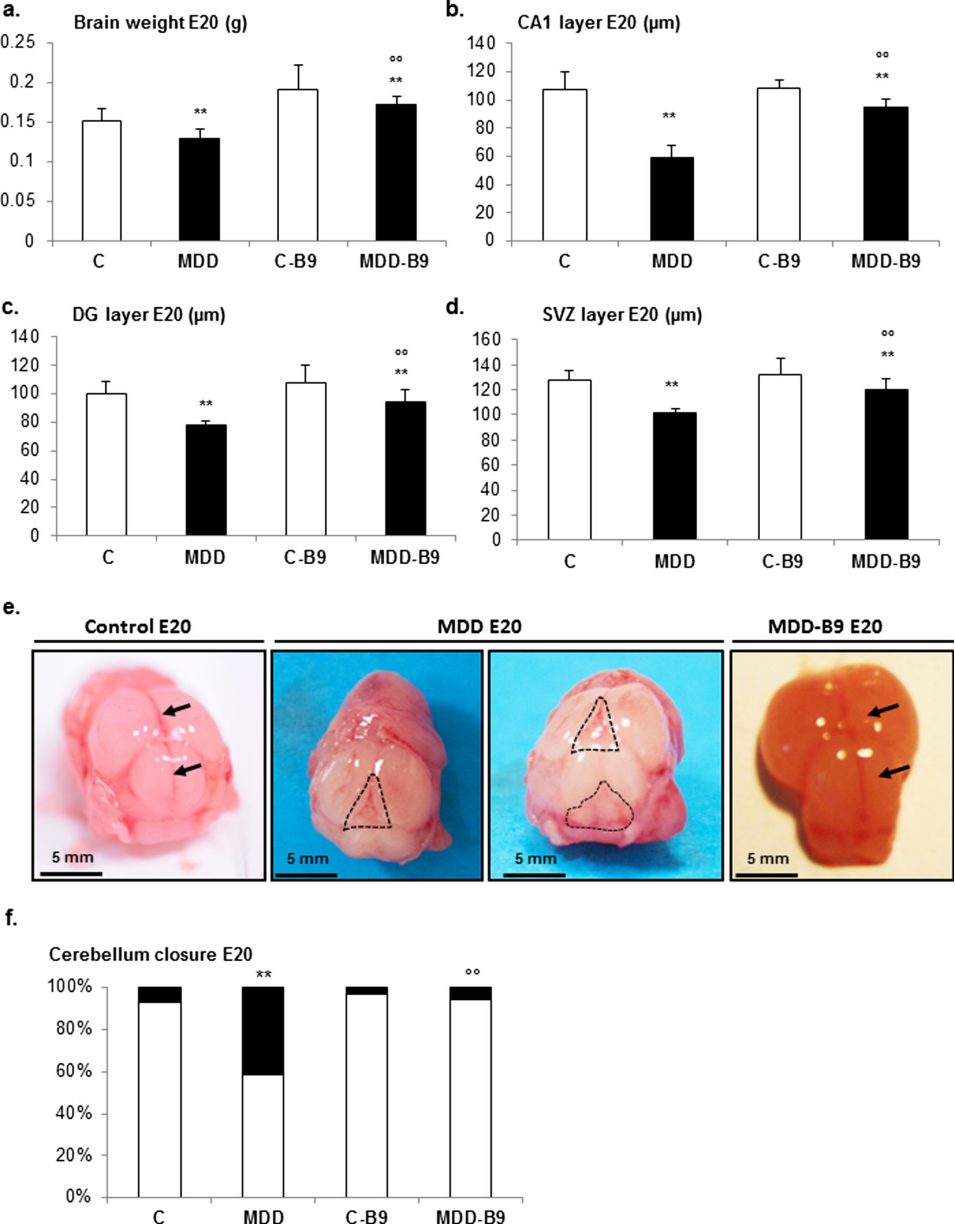
Brain defects associated with methyl donor deficiency in rat fetuses: influence of folic acid supplementation. **a** Brain weight in control (*C*), methyl donor-deficient (*MDD*), supplemented control (*C-B9*), and supplemented-deficient (*MDD-B9*) fetuses at E20 (15 ≤ *n* ≤ 29). Statistically significant differences between control and MDD rats, **P < 0.01, and between MDD and supplemented MDD, °°*P* < 0.01. **b–d** Thicknesses of hippocampal CA1 pyramidal cell layer, granular cell layer of the dentate gyrus (*DG*), and subventricular zone (*SVZ*) at E20 (12 ≤ *n* ≤ 21). Statistically significant differences between control and MDD rats, **P < 0.01, and between MDD and supplemented MDD, °°*P* < 0.01. **e** Improper interhemispheric junction and cerebellar vermis closure (delineated by *dotted line areas*) in MDD rat fetuses at E20 and beneficial effects of folic acid supplementation (*arrows*). **f** Prevalence of defective closure of the cerebellar vermis in the various experimental groups. Statistically significant differences between control and MDD rats, **P < 0.01, and between MDD and supplemented MDD, °°*P* < 0.01

While brain weight at E20 was reduced in the same proportions as body weight (about 15 %) following exposure to methyl donor deficiency, thicknesses of brain layers such as the hippocampal CA1 pyramidal layer, the granular cell layer of the dentate gyrus, and the neurogenic subventricular zone (SVZ) were more dramatically affected (from 25 to 40 %; Fig. 2a–d). Again, belated supplementation allowed significant reduction of these defects. Importantly, deficiency was associated, at this developmental stage, with delayed closure of the cephalic parts of the neural tube, as reflected by improper interhemispheric junction and open cerebellar vermis that could be largely prevented by folic acid supplementation (Fig. 2e). As illustrated by Fig. 2f, the occurrence of open cerebellar vermis at E20 varied from 41 % in deficient fetuses to ∼3 % in controls and supplemented counterparts.

### Methyl Donor Deficiency Increases Expression Levels of Let-7 and miR-34: Reversion by Folic Acid Supplementation

**Fig. 3:**
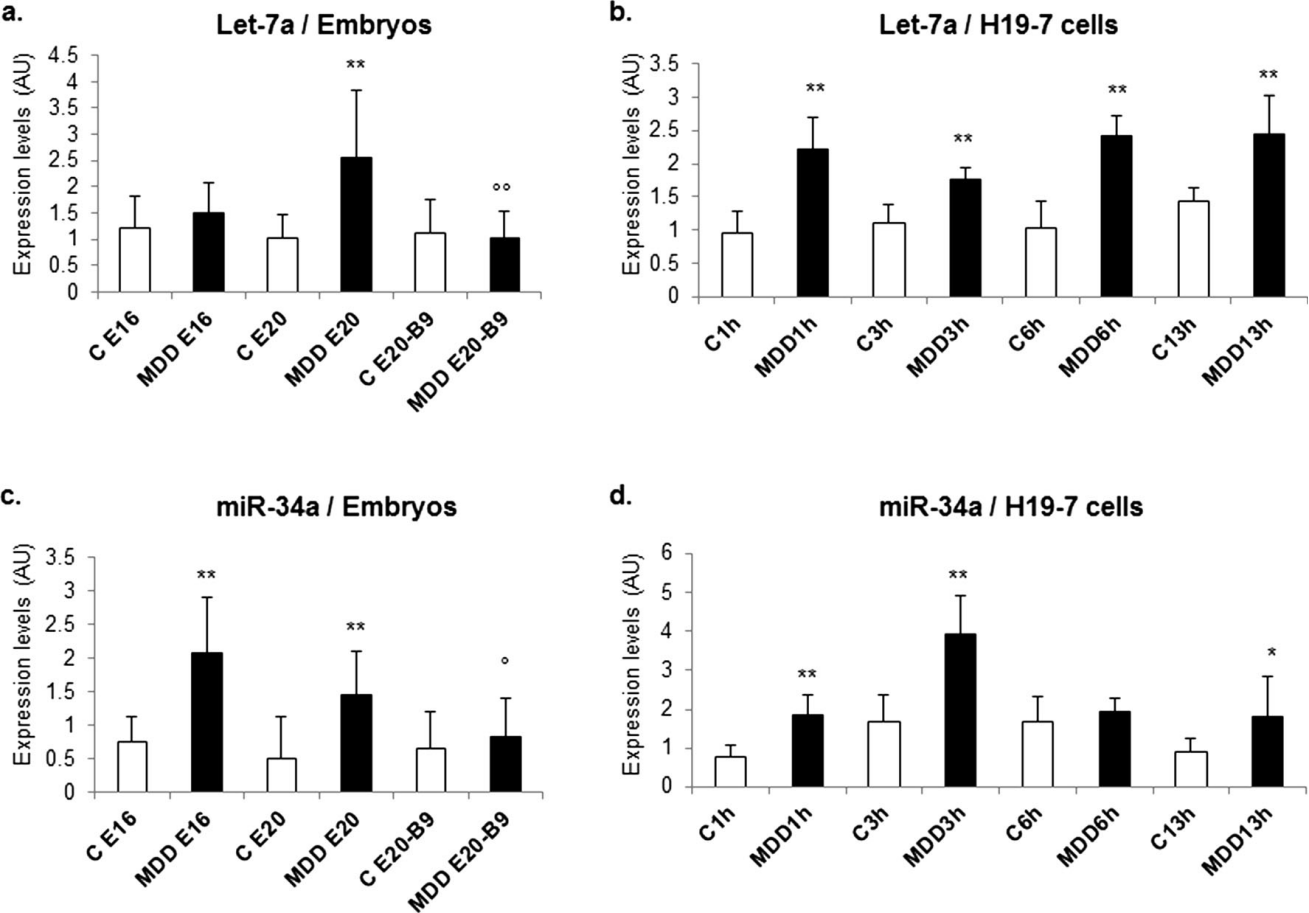
Effects of methyl donor deficiency on the expression of let-7 and miR-34: influence of folic acid supplementation. **a** Expression levels of let-7 in arbitrary units (*AU*) in the midbrains of control (*C*) and deficient (*MDD*) rat embryos at E16 and E20, and effects of folic acid (B9) supplementation. **b** Expression levels in control (*C*) and B9-deficient (*MDD*) H19-7 hippocampal cells after induction of their differentiation. **c** Expression levels of miR-34 in the midbrains of control and deficient rat embryos, and effects of folic acid supplementation. **d** Expression levels in control and B9-deficient H19-7 cells. All data are reported as means ± S.D. (12 ≤ *n* ≤ 24). Statistically significant differences between control and MDD, *P < 0.05 and**P < 0.01, and between MDD and supplemented MDD, °*P* < 0.05 and °°*P* < 0.01

When studied by TaqMan RT-qPCR, let-7a expression patterns showed a significant increase (by 2.5-fold) in the midbrain extracts from E20 deficient fetuses, an effect that was reversed by folic acid supplementation (Fig. 3a). Let-7 levels were increased by the same order of magnitude in vitro in differentiating hippocampal progenitors lacking folate (Fig. 3b). Similar observations could be made regarding the expression patterns of miR-34a (Fig. 3c, d). This was confirmed by in situ hybridization in various brain areas including the hippocampus, the cerebral cortex, and the cerebellum (Supplementary Fig. S1 and S2).

### Early Methyl Donor Deficiency Alters the Expression Pattern of a Wide Range of Genes Influenced by Let-7 and miR-34 and Involved in Various Aspects of Development

**Fig. 4:**
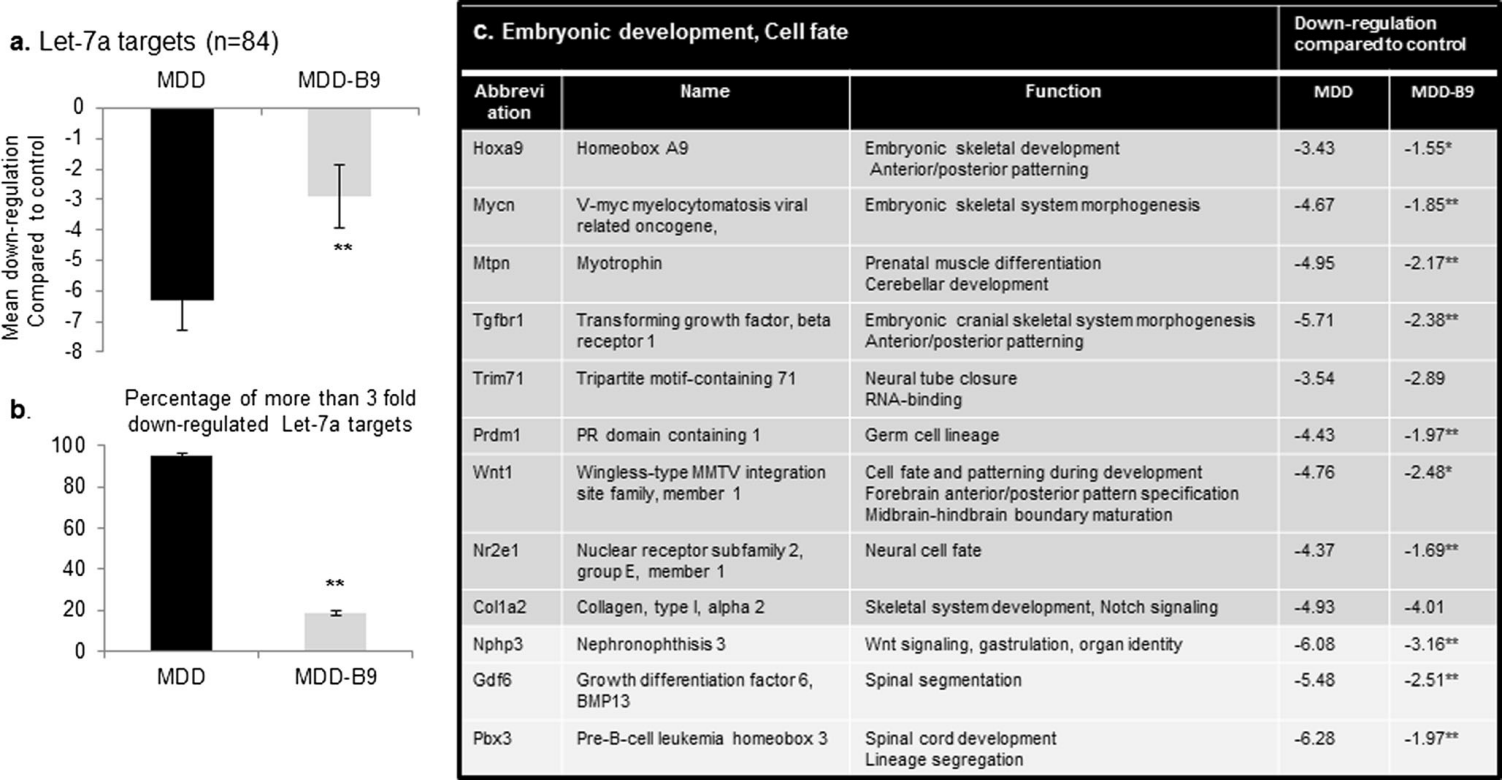
Transcriptional alteration of relevant experimentally documented or bioinformatically predicted gene targets of let-7a in fetal brain under methyl donor deficiency: effects of folic acid supplementation. qPCR analysis was performed by means of Rat microRNA Targets RT^2^ Profiler PCR Arrays (Qiagen) monitoring 84 specific genes. Biological samples were obtained from the brains of two to three E20 fetuses for each condition and were tested in duplicate in separate plates. **a** Mean downregulation (± S.D.) in methyl donor-deficient (MDD) and supplemented-deficient (MDD-B9) fetuses. **b** Percentage of genes downregulated by more than 3-fold. **c** Downregulation of selected target genes involved in embryonic development and cell fate. Statistically significant differences between MMD and MDD-B9: *P < 0.05, **P < 0.01

**Fig. 5:**
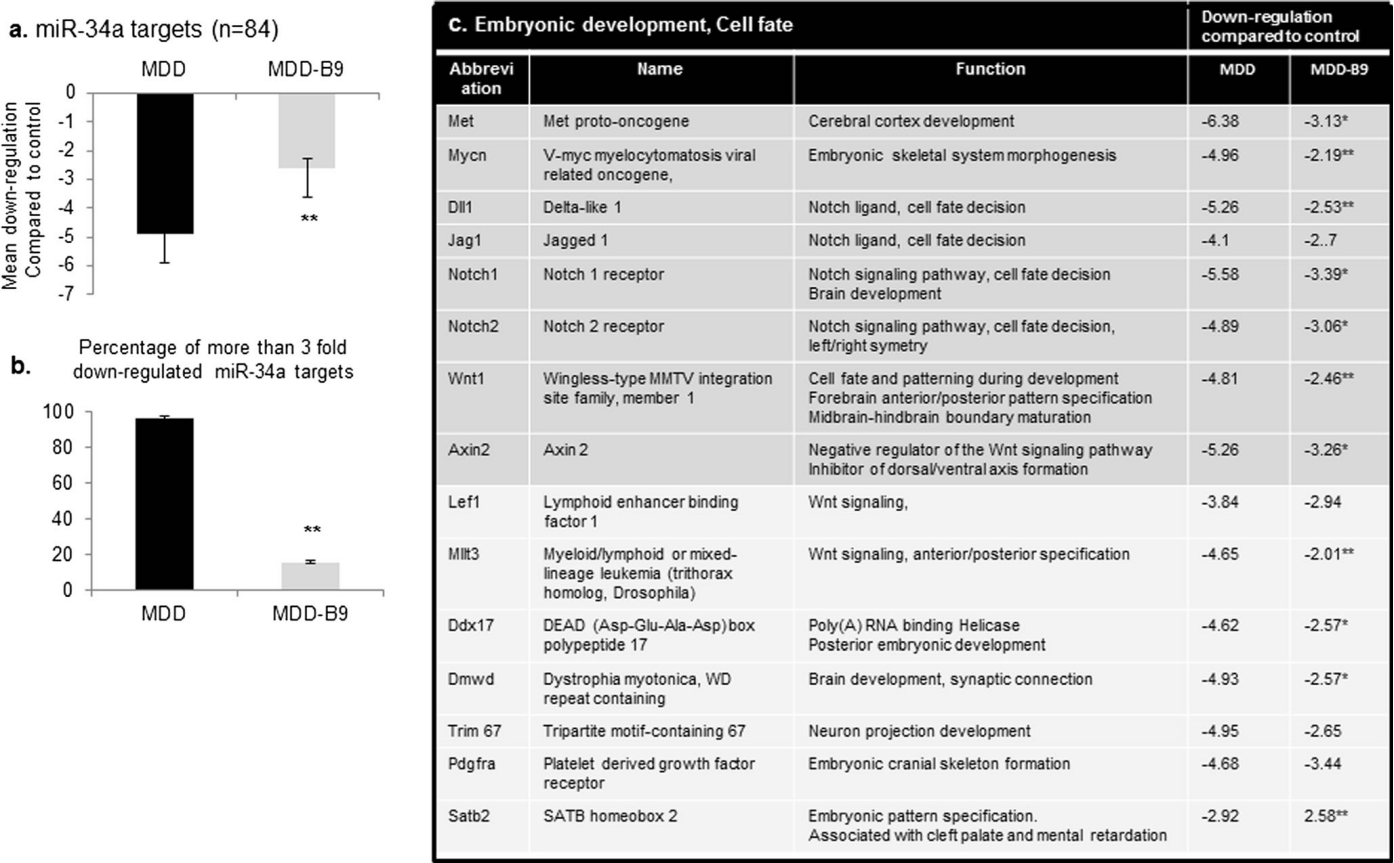
Transcriptional alteration of relevant experimentally documented or bioinformatically predicted gene targets of miR-34a in fetal brain under methyl donor deficiency: effects of folic acid supplementation. qPCR analysis was performed by means of Rat microRNA Targets RT^2^ Profiler PCR Arrays (Qiagen) monitoring 84 specific genes. Biological samples were obtained from the brains of two to three E20 fetuses for each condition and were tested in duplicate in separate plates. **a** Mean downregulation (± S.D.) in methyl donor-deficient (MDD) and supplemented-deficient (MDD-B9) fetuses. **b** Percentage of genes downregulated by more than 3-fold. **c** Downregulation of selected target genes involved in embryonic development and cell fate. Statistically significant differences between MMD and MDD-B9: *P < 0.05, **P < 0.01

As shown by Figs. 4 and 5, microarray approach revealed that methyl donor deficiency was associated with an overall decreased expression of known or predicted target genes. More specifically, the average downregulation of let-7a targets was above 6-fold in brain tissues from deficient fetuses, 95 % being downregulated by more than 3-fold. Among them, 12 genes were identified to participate to embryonic development and cell fate (Fig. 4c), while others are involved in cell migration, cytoskeleton organization, cell cycle, and RNA trafficking, as indicated in supplementary data (Supplementary Table S1). Folic acid supplementation reduced significantly the dysregulation of almost all target genes affected. Regarding miR-34a, the average downregulation of its targets was ∼5-fold in deficient samples, 96 % being downregulated by more than 3-fold (Fig. 5a, b). Fifteen genes are related to embryonic development and cell fate (Fig. 5c). Others are involved in cell migration, axon guidance, cell cycle, and vesicular trafficking (Supplementary Table S2). Again, folic acid supplementation was able to ameliorate gene expression.

### Methyl Donor Deficiency Affects Protein Expression Levels of Known Downstream Pathways of Let-7 and miR-34: Reversion by Folic Acid Supplementation

**Fig. 6:**
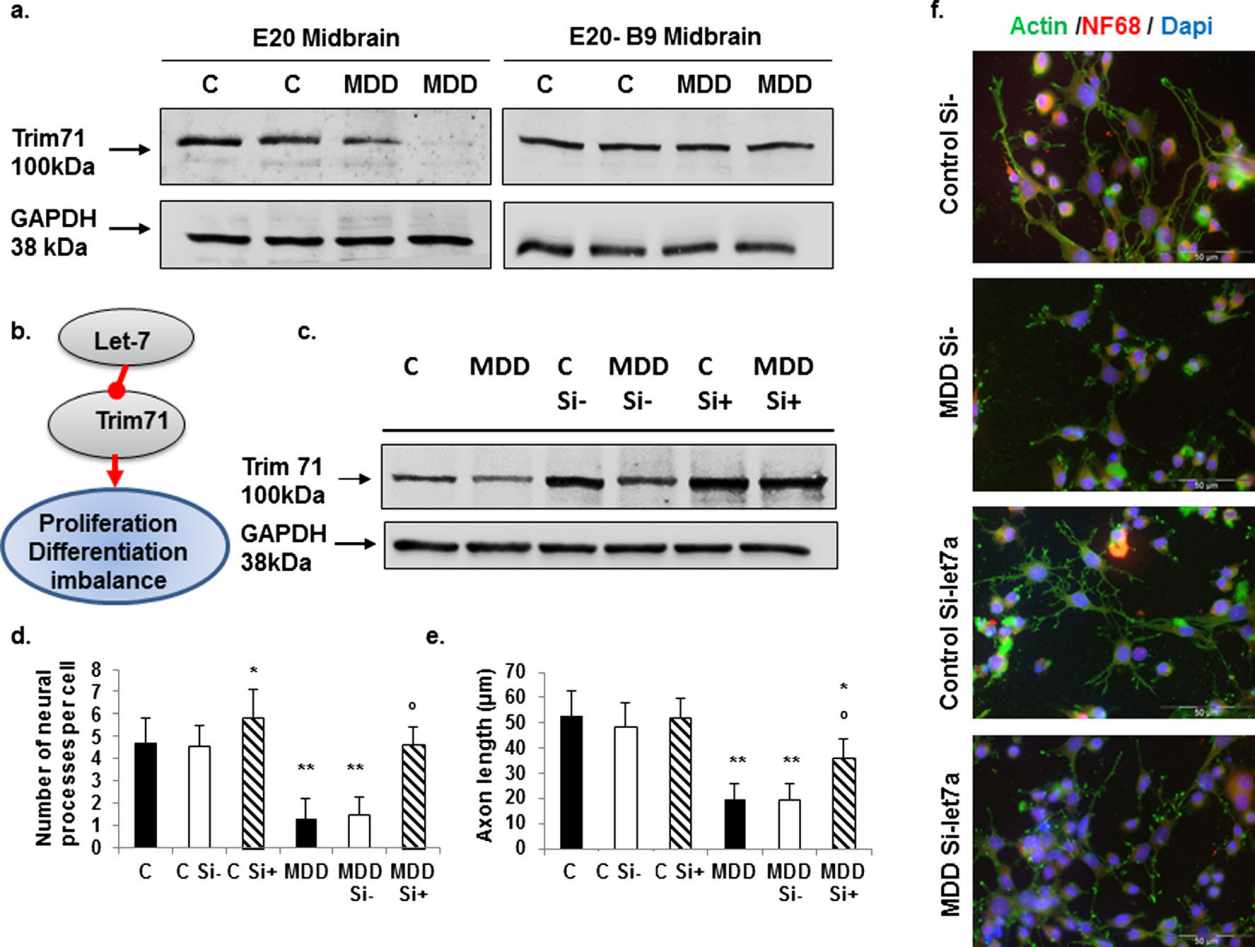
Effects of methyl donor deficiency and folic acid supplementation on Trim71, a target of let-7. Consequences of silencing let-7a on differentiating H19-7 cells. **a** Western blot analyses of Trim71 expression in the midbrain of control (*C*) and deficient (*MDD*) E20 fetuses and effect of acid folic (B9) supplementation. Similar findings were obtained from three other experiments using tissue extracts from distinct embryonic brains. **b** Summary of events involved in the consequences of methyl donor deficiency in the rat brain (see the “Discussion” section). **c** Expression levels of Trim71 and effects of let-7 siRNA in control (*C*) and folate-deficient (MDD) H19-7 cells at 13 h after induction of differentiation (Si− = non-targeting siRNA, Si+ = let-7-targeted siRNA). Expression patterns are representative of four separate series of Western blots. **d** Average number of cell processes per cell. **e** Mean axon length. For **d** and **e**: statistically significant difference with the respective control: *P < 0.05 and **P < 0.01; statistically significant difference between Si− and Si+: °*P* < 0.05 (*n* = 5). **f** Representative influence of let-7 siRNA on cell morphology at 13 h after induction of differentiation. Cells are colabeled with antibodies against actin (*green*) and NF68 (*red*) and their nuclei counterstained by Dapi (*blue*)

We chose to investigate Trim71, which is a key effector of the let-7 microRNA pathway, that promotes cell proliferation and inhibits differentiation to control various developmental processes [45]. Western blot analysis showed that the expression level of Trim71 in the fetus midbrain was repressed at E20 following methyl donor deficiency, whereas it was restored to normal level following folic acid supplementation (Fig. 6a).

**Fig. 7:**
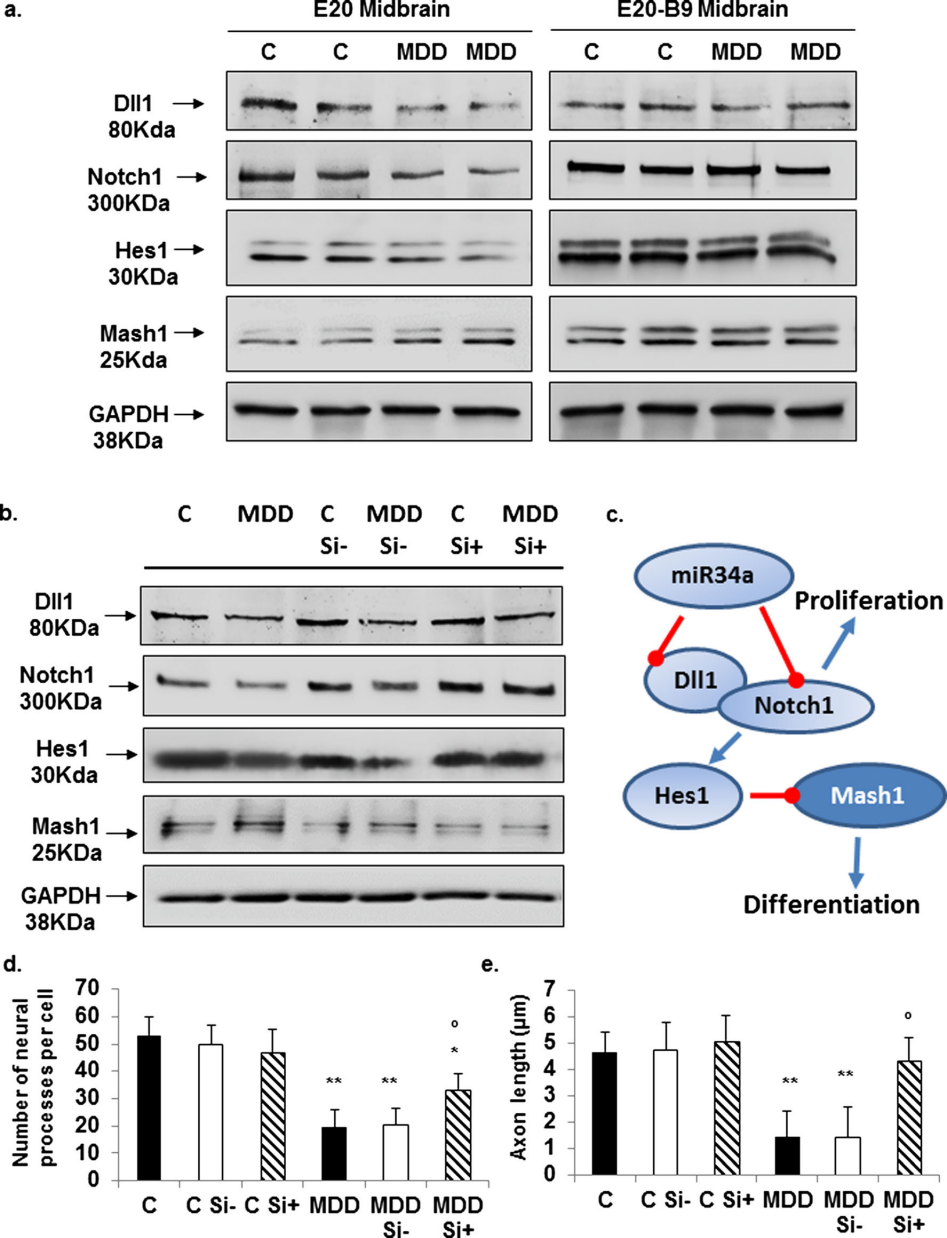
Effects of methyl donor deficiency and folic acid supplementation on Notch signaling proteins, targets of miR-34. Consequences of silencing miR-34a on differentiating H19-7 cells. **a** Western blot analyses of Notch signaling proteins expression in the midbrain of control (*C*) and deficient (*MDD*) E20 fetuses and effect of acid folic (B9) supplementation. Similar findings were obtained from three other experiments using tissue extracts from distinct embryonic brains. **b** Expression levels of Notch signaling proteins and effects of miR-34 siRNA in control (C) and folate-deficient (MDD) H19-7 cells at 13 h after induction of differentiation (Si− = non-targeting siRNA, Si+ = miR-34-targeted siRNA). Expression patterns are representative of four separate series of Western blots. **c** Summary of events involved in the consequences of methyl donor deficiency in the rat brain (see the “Discussion” section). **d** Average number of cell processes per cell. **e** Mean axon length. For **d** and **e**: statistically significant difference with the respective control: *P < 0.05 and**P < 0.01; statistically significant difference between Si− and Si+: °*P* < 0.05 (*n* = 5)

The Notch receptor ligand delta-like 1 (dll1) is a target of miR-34, and during morphogenesis of the central nervous system, differentiation of neural progenitors is known to be inhibited by hairy and enhancer of split homolog 1 (Hes1), whereas it is stimulated by mammalian achaete-scute complex homolog 1 (Mash1) [46]. We, therefore, focused our study more specifically on Notch signaling pathway. Under deficiency conditions, midbrain expression levels of dll1, Notch1, and Hes1 were noticeably reduced, whereas the expression of Mash1 was more elevated (Fig. 7a). When folic acid supplementation was applied, the expression of the various proteins in deficient fetuses was similar to controls.

### Silencing Let-7a and miR-34a Restores Their Related Pathways and Contributes to Rescue Cells Exposed to Folate Deficiency

Let-7 siRNA that repeatedly inhibited the expression of let-7a by >85 % after quantification by TaqMan RT-qPCR increased Trim71 levels in both control and folate-deficient H19-7 cells at 13 h after induction of their differentiation (Fig. 6c). The detrimental role of increased let-7a in folate-deficient progenitors was further supported by cell morphology of differentiating progenitors (Fig. 6d–f). Indeed, folate deficiency was accompanied at 13 h of differentiation by a strong reduction of the number and length of neural processes, as previously documented [32]. The downregulation of let-7a by siRNA led to a noticeable restoration of these processes. Although the treatment by let-7 siRNA slightly modified cell characteristics in control progenitors, reflecting increased branching projections, both the number of neurites per cell and the axon length, were significantly augmented in folate-deficient cells treated by let-7 siRNA as compared with those treated by non-targeting siRNA (Fig. 6d–f).

Although the use of non-targeting siRNA (Si−) had no patent effect on the expression of Notch signaling proteins in differentiating cells, silencing miR-34a increased significantly the expression of ddl1, Notch1, and Hes1, while protein amounts of Mash1 were reduced (Fig. 7b). As for let-7, miR-34 siRNA had beneficial effects on the morphological characteristics of deficient cells (Fig. 7d, e and Supplementary Fig. S3).

### Methyl Donor Deficiency Impairs Basic Cognitive Performances in the Homing Test which Are Ameliorated by Folic Acid Supplementation

**Fig. 8:**
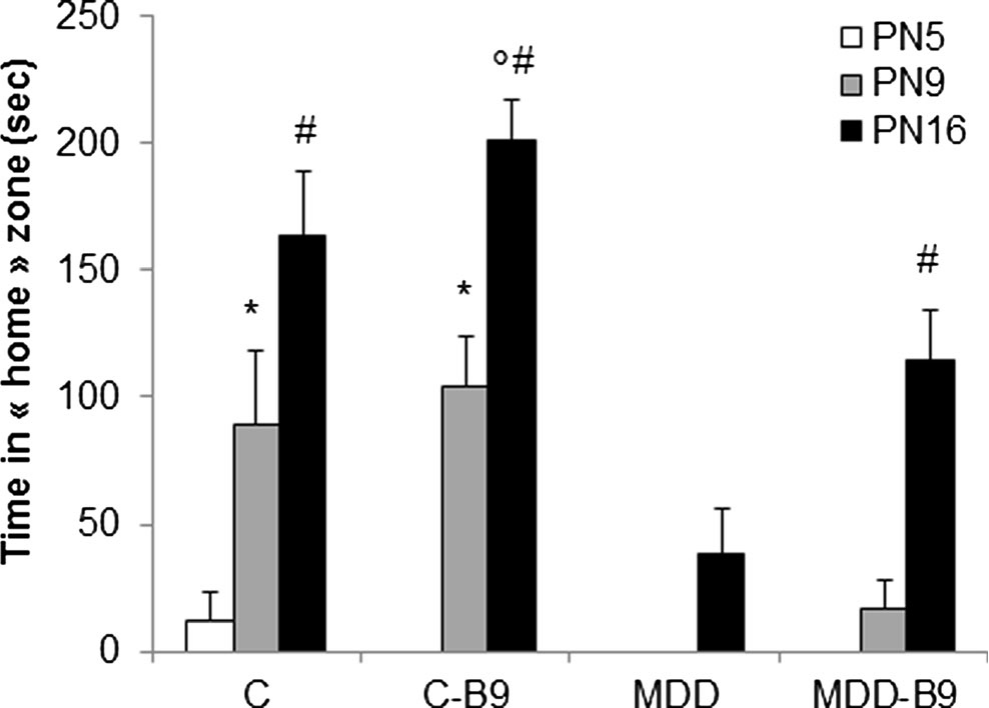
Effects of methyl donor deficiency and folic acid supplementation on the homing scores of rat pups. Tests were performed on postnatal (PN) days 5, 9, and 16 in the four experimental groups corresponding to controls (*C*) and methyl donor-deficient (*MDD*) pups whose mothers received either vehicle (i.e., milk) or folic acid (B9) supplementation (11 ≤ *n* ≤ 40). Data are reported as time (in seconds) spent in the home area ± S.D. At PN 9, statistically significant difference from MDD with or without B9 supplementation: * *P* < 0.01. At PN 16, statistically significant difference from MDD with or without B9 supplementation: ° *P* < 0.01, and from MDD without B9 supplementation: ^#^ *P* < 0.05

In the early postnatal period corresponding to 5–16 days of age, control rat pups displayed a strong acquisition of the “home” signals, as shown by a progressive increase of the time spent in the home area (Fig. 8). This reflects a learning process of environmental cues. By contrast, methyl donor-deficient pups did not exhibit such an acquisition, with no measurable time spent in the home zone at postnatal days 5 and 9, and reduced time at postnatal day 16. Although maternal folic acid supplementation had no patent effects in control pups, it significantly improved the performance of deprived pups who showed a 2.5-fold increase of the time spent in the home area as compared to the non-supplemented deficient group (*p* < 0.05).

## Discussion

Folate deficiency is known to affect fetal and neonatal brain development and can result in various functional deficits (see [47] for recent review). Developmental disorders are mediated by a wide range of underlying cellular mechanisms associated with vitamin deficiency. In the present study, we provide the first evidence that the epigenetic overexpression of let-7a and miR-34a, along with the disruption of their related pathways, would be key players in the deleterious effects of early methyl donor deficiency on the anatomical and functional development of the central nervous system. While it cannot fully prevent early-occurring NTDs such as spina bifida, maternal supplementation with folic acid during the period corresponding to the last trimester of pregnancy in women appeared to help preserve a normal development, at least partly through restoring let-7 and miR-34 normal expression.

In our animal model, maternal exposure to methyl donor deficiency is associated with various developmental abnormalities, such as spontaneous abortion, intrauterine growth retardation, congenital malformations, and delayed ossification. Underdeveloped skeleton has been already reported in relationship with low-folate status in the periconceptional period or following low-paternal dietary folate [48]. Also, there was a high prevalence of apparent NTDs. These include defective closure of the posterior neural tube as reflected by “twisted” tail and open spinal canal, both signs being suggestive of spina bifida. Such developmental disorders associated with folate and/or vitamin B12 deficiency have been largely reported [11, 12, 49]. In addition to a global atrophy of the brain in deficient pups, we frequently observed improper interhemispheric junction and unclosed cerebellar vermis, suggesting developmental anomalies of the cephalic part of the neural tube. The embryonic development of NTDs is complex, with diverse mechanisms operating at different levels of the body axis. Although homocysteine was shown to be embryotoxic, it did not appear to be directly responsible for NTDs [50], and the mechanisms underlying the effect of maternal B vitamin status on neural tube development remain elusive.

Neurulation, which is conventionally divided into primary and secondary phases, is a fundamental event of embryogenesis that culminates in the formation of the neural tube, the precursor of the brain and spinal cord, and neural tube closure is an essential step in the development of the central nervous system [51]. The neuroepithelium is entirely proliferative during neurulation. Cells begin to exit the cell cycle, with the onset of neuronal differentiation, only after neural tube closure is complete. Accurate control of proliferation, differentiation, and apoptosis of neural cells is crucial for proper development and maturation of the nervous system. This requires the temporal expression of specific gene sets, most of them being influenced by epigenetic processes [52, 53]. In this respect, regulation of gene expression by microRNAs is proving to be essential for the neurogenic process [54]. Under the conditions of methyl donor deficiency, we observed a disruption of the expression of let-7a and miR-34a and their related pathways. Among the microRNAs relevant to vertebrate nervous system development, the highly conserved let-7a microRNA would constitute a key regulator of neural cell proliferation and differentiation [55] and has been tightly associated with the occurrence of NTDs [29]. Depending on the experimental models used, it was reported that let-7a could act through various pathways involving the participation of transcription factors such as Abrupt, Sox2, Tlx, or cell cycle regulators such as CDK/Cyclin complexes [56, 57], contributing to the overall effect of let-7 on increasing the number of cells in the G1 phase of the cell cycle. In the present study, we chose to further investigate the well-established target of let-7, Trim71 (also called Lin41), which is required for embryonic development and proper neural tube closure [29, 58]. According to the study by Maller Schulman et al. [29] in the mouse, Trim71 is necessary for initiation and correct closure of the neural tube at the midbrain/hindbrain border. Belonging to the family of Trim-NHL ubiquitin ligases, Trim71 is highly expressed in undifferentiated cells, such as embryonic stem cells, but becomes rapidly downregulated upon differentiation, in response to the rise of let-7 levels [59]. It was reported to cooperate with microRNAs to repress Cdkn1a expression and promote embryonic cell proliferation [60]. The data available suggest a role for Trim71 in the maintenance of stem cell identity and/or inhibition of premature differentiation. In our experimental models of methyl donor deficiency, we observed a strong increase in let-7a expression, especially in rat pups at the E20 stage and in differentiating progenitors, along with significantly lower levels of Trim71 protein. These results are consistent with the relatively high prevalence of anomalies of the cephalic part of the neural tube depicted in E20 rat pups. Regarding miR-34a, its expression levels were also found to be significantly higher under methyl donor deficiency. Described as belonging to a “late-brain development” expression cluster in the mouse [61], miR-34a regulates numerous target genes involved in cell cycle, apoptosis, differentiation, and neuron maintenance [62]. More specifically, an increase in the expression of miR-34a leads to a decrease of dll1, which mediates its effects by binding to the Notch receptor, causing its proteolytic cleavage and release of the Notch intracellular domain [63]. Notch is known to play a role in the developing central nervous system by inhibiting neuronal differentiation and maintaining neural precursor cells. Hes1, a major downstream target of Notch, suppresses neuronal differentiation by repressing expression of, among other targets, the activator-type bHLH gene *Mash1* [64]. In the present study, all the above-mentioned members of the Notch signaling pathway were dramatically affected by methyl donor deficiency. This would finally result in decreased cell proliferation and premature neuronal differentiation [65]. Taken together, our data therefore suggest that the alterations observed in let-7 and miR-34 pathways in response to methyl donor deficiency may participate to a disruption of the proliferation/differentiation balance, resulting in improper development of the central nervous system, and influencing the occurrence of NTDs. As pointed out by Copp et al. [66], premature differentiation in the neuroepithelium might render the neural plate mechanically inflexible and prevent dorsolateral bending, interfere with the release of neural crest cells or inhibit the adhesion process that is necessary for neural fold fusion. Furthermore, overexpression of miR-34a has been reported to alter neurite outgrowth and synaptogenesis, with functional consequences, in particular at the electrophysiological level [67]. Such observations are consistent with our previous findings in developing neuroprogenitors showing that folate deprivation markedly affects cell polarity, neurite outgrowth, vesicular transport, and synaptic function and plasticity [32]. Finally, the methyl donor deficiency-associated poorer scores recorded in the homing test at postnatal days 5–16 are in good correlation with the observation of persisting atrophy of some brain cell layers, notably in the hippocampus, in rat pups exposed to the same conditions of B vitamin deficiency as in the present study [14], suggesting long-lasting functional defects.

Our experimental conditions reduced methyl donor availability and affected the one-carbon metabolism, and thus, methylation capacities, as previously documented both in the rat progeny [30, 43, 68] and in H19-7 neuroprogenitors [32]. Periconceptional/gestational administration of folic acid is recognized for its beneficial effects, especially for the prevention of NTDs. However, questions remain about the optimal dose and appropriate periodicity of folic acid supplementation. The U.S. Public Health Service and CDC recommend that all women of childbearing age ingest 0.4 mg folic acid daily. WHO guidelines also recommend 0.4 mg per day for all women between 15 and 45 years of age, whereas women with history of NTD are encouraged to take 4 mg during the first 3 months of pregnancy. Folate deficiency during late gestation has been shown to decrease progenitor cell proliferation and increases apoptosis in fetal mouse brain [69, 70]. In addition, it has been reported that differences in birth weight and neurocognitive development related to environmental factors are most likely initiated during the second and third trimesters of pregnancy [71]. As a consequence of our protocol of belated supplementation, metabolic features were ameliorated, and we observed a reduction of structural and functional (homing test) defects taking place during the peak of brain maturation in the perinatal period. Most importantly, folic acid supplementation helped restoring the levels of let-7 and miR-34 and their respective targets.

As recently reviewed by Imbard et al. [22], several mechanisms have been proposed to account for the role of folate and vitamin B12 during embryogenesis. They include decreased oxidative stress and increased nucleotide biosynthesis, facilitating DNA replication. Since both vitamins are involved in the methylation pathway, several authors have pointed out that disturbed methylation and related epigenetic changes could be responsible for developmental abnormalities and birth defects [19–21]. This is supported by the deleterious effects of polymorphisms in genes involved in the methylation pathway such as *methylenetetrahydrofolate reductase* (*MTHFR*), coding for a key enzyme of folate metabolism implied in homocysteine remethylation [72, 73], and whose activity is affected in our animal model [74]. Interestingly, MTHFR is a predicted target of miR-34a [27]. An increase in miR-34a would lead to a decrease in MTHFR expression, reducing methionine amounts to finally decrease methylation reactions and increase homocysteine levels.

In conclusion, we showed that methyl donor deficiency was associated with enhanced expression of let-7a and miR-34a, with subsequent alterations of their development regulatory targets such as Trim71 and Notch signaling partners. The latter data are consistent with the previous demonstrations that folic acid is able to stimulate Notch signaling, including Hes1 transcription, and promotes cell proliferation in neural stem cells [75–77]. Taken together, the positive impact observed after folic acid supplementation and following siRNA strategy, along with the associated normalization of let-7a and miR-34a, strengthen the potential role of these microRNAs and their related signaling pathways in the developmental defects consecutive to gestational methyl donor deficiency. By using the microarray approach, the identification of new putative target genes affected in response to methyl donor deficiency via let-7 and miR-34 warrants further investigations. Finally, our data support late gestational folate supplementation in at risk women, as we report for the first time its beneficial role on brain maturation in the progeny. According to the “fetal programming” hypothesis, gestational exposure to appropriate concentrations of methyl donors may help reduce the susceptibility to certain diseases in adulthood by favorably influencing epigenetic profiles [78].

## Electronic supplementary material

Fig. S1 Effects of methyl donor deficiency and folic acid supplementation on the expression of let-7a as depicted by in situ hybridization in the hippocampus, cerebellum and cerebral cortex from E20 fetuses (PDF 230 kb)

Fig. S2 Effects of methyl donor deficiency and folic acid supplementation on the expression of miR-34a as depicted by in situ hybridization in the hippocampus, cerebellum and cerebral cortex from E20 fetuses (PDF 201 kb)

Fig. S3 Consequences of miR-34a silencing on the morphology of control and folate-deficient (MDD) H19–7 cells at 13 h after induction of their differentiation (Si- = non-targeting siRNA, Si-miR-34a = miR-34a targeted siRNA). Cells are colabeled with antibodies against actin (green) and NF68 (red) and their nuclei counterstained by Dapi (blue) (PDF 150 kb)

Table S1 Downregulation of let-7a gene targets, according to their known functions, in methyl donor deficient (MDD) and supplemented deficient (MDD-B9) brain fetuses. Statistically significant differences between MMD and MDD-B9: *P < 0.05, **P < 0.01 (PDF 176 kb)

Table S2 Downregulation of miR-34a gene targets, according to their known functions, in methyl donor deficient (MDD) and supplemented deficient (MDD-B9) brain fetuses. Statistically significant differences between MMD and MDD-B9: *P < 0.05, **P < 0.01 (PDF 175 kb)

## References

[1] Aranda P, Agirre X, Ballestar E, Andreu EJ, Román-Gómez J, Prieto I, Martín-Subero JI, Cigudosa JC (2009). Epigenetic signatures associated with different levels of differentiation potential in human stem cells. PLoS One.

[2] Cantone I, Fisher AG (2013). Epigenetic programming and reprogramming during development. Nat Struct Mol Biol.

[3] Zhang TY, Meaney MJ (2010). Epigenetics and the environmental regulation of the genome and its function. Annu Rev Psychol.

[4] Lo CL, Zhou FC (2014). Environmental alterations of epigenetics prior to the birth. Int Rev Neurobiol.

[5] McMillen IC, MacLaughlin SM, Muhlhausler BS, Gentili S, Duffield JL, Morrison JL (2008). Developmental origins of adult health and disease: the role of periconceptional and foetal nutrition. Basic Clin Pharmacol Toxicol.

[6] Attig L, Gabory A, Junien C (2010). Nutritional developmental epigenomics: immediate and long-lasting effects. Proc Nutr Soc.

[7] Guéant JL, Caillerez-Fofou M, Battaglia-Hsu S, Alberto JM, Freund JN, Dulluc I, Adjalla C, Maury F (2013). Molecular and cellular effects of vitamin B12 in brain, myocardium and liver through its role as co-factor of methionine synthase. Biochimie.

[8] Guéant JL, Namour F, Guéant-Rodriguez RM, Daval JL (2013). Folate and fetal programming: a play in epigenomics?. Trends Endocrinol Metab.

[9] Hoffman DR, Cornatzer WE, Duerre JA (1979). Relationship between tissue levels of S-adenosylmethionine, S-adenylhomocysteine, and transmethylation reactions. Can J Biochem.

[10] McKay JA, Williams EA, Mathers JC (2004). Folate and DNA methylation during in utero development and aging. Biochem Soc Trans.

[11] Kirke PN, Molloy AM, Daly LE, Burke H, Weir DG, Scott JM (1993). Maternal plasma folate and vitamin B12 are independent risk factors for neural tube defects. Q J Med.

[12] Molloy AM, Kirke PN, Brody LC, Scott JM, Mills JL (2008). Effects of folate and vitamin B12 deficiencies during pregnancy on fetal, infant, and child development. Food Nutr Bull.

[13] Black MM (2008). Effects of vitamin B12 and folate deficiency on brain development in children. Food Nutr Bull.

[14] Kerek R, Geoffroy A, Bison A, Martin N, Akchiche N, Pourié G, Helle D, Guéant JL (2013). Early methyl donor deficiency may induce persistent brain defects by reducing Stat3 signaling targeted by miR-124. Cell Death Dis.

[15] Bailey LB (2004). Folate and vitamin B12 recommended intakes and status in the United States. Nutr Rev.

[16] De-Regil LM, Fernández-Gaxiola AC, Dowswell T, Peña-Rosas JP (2010). Effects and safety of periconceptional folate supplementation for preventing birth defects. Cochrane Database Syst Rev.

[17] Czeizel AE, Dudás I, Paput L, Bánhidy F (2011). Prevention of neural-tube defects with periconceptional folic acid, methylfolate, or multivitamins?. Ann Nutr Metab.

[18] Fekete K, Berti C, Trovato M, Lohner S, Dullemeijer C, Souverein OW, Cetin I, Decsi T (2012). Effect of folate intake on health outcomes in pregnancy: a systematic review and meta-analysis on birth weight, placental weight and length of gestation. Nutr J.

[19] Afman LA, Blom HJ, Drittij MJ, Brouns MR, van Straaten HW (2005). Inhibition of transmethylation disturbs neurulation in chick embryos. Brain Res Dev Brain Res.

[20] Dunlevy LP, Burren KA, Mills K, Chitty LS, Copp AJ, Greene ND (2006). Integrity of the methylation cycle is essential for mammalian neural tube closure. Birth Defects Res.

[21] Greene ND, Stanier P, Moore GE (2011). The emerging role of epigenetic mechanisms in the etiology of neural tube defects. Epigenetics.

[22] Imbard A, Benoist JF, Blom HJ (2013). Neural tube defects, folic acid and methylation. Int J environ res. Public Health.

[23] Kapsimali M, Kloosterman WP, de Bruijn E, Rosa F, Plasterk RH, Wilson SW (2007). MicroRNAs show a wide diversity of expression profiles in the developing and mature central nervous system. Genome Biol.

[24] Fineberg SK, Kosik KS, Davidson BL (2009). MicroRNAs potentiate neural development. Neuron.

[25] Yi R, Fuchs E (2011). MicroRNAs and their roles in mammalian stem cells. J Cell Sci.

[26] Petri R, Malmevik J, Fasching L, Åkerblom M, Jakobsson J (2014). miRNAs in brain development. Exp Cell Res.

[27] Shookhoff JM, Gallicano GI (2010). A new perspective on neural tube defects: folic acid and microRNA misexpression. Genesis.

[28] Sato F, Tsuchiya S, Meltzer SJ, Shimizu K (2011). MicroRNAs and epigenetics. FEBS J.

[29] Maller Schulman BR, Liang X, Stahlhut C, DelConte C, Stefani G, Slack FJ (2008). The let-7 microRNA target gene, Mlin41/Trim71 is required for mouse embryonic survival and neural tube closure. Cell Cycle.

[30] Blaise SA, Nédélec E, Schroeder H, Alberto JM, Bossenmeyer-Pourié C, Guéant JL, Daval JL (2007). Gestational vitamin B deficiency leads to homocysteine-associated brain apoptosis and alters neurobehavioral development in rats. Am J Pathol.

[31] Daval JL, Blaise S, Guéant JL (2009). Vitamin B deficiency causes neural cell loss and cognitive impairment in the developing rat. Proc Natl Acad Sci USA.

[32] Akchiche N, Bossenmeyer-Pourié C, Kerek R, Martin N, Pourié G, Koziel V, Helle D, Alberto JM (2012). Homocysteinylation of neuronal proteins contributes to folate deficiency-associated alterations of differentiation, vesicular transport, and plasticity in hippocampal neuronal cells. FASEB J.

[33] Ducros V, Belva-Besnet H, Casetta B, Favier A (2006). A robust liquid chromatography tandem mass spectrometry method for total plasma homocysteine determination in clinical practice. Clin Chem Lab Med.

[34] Chery C, Barbe F, Lequere C, Abdelmouttaleb I, Gerard P, Barbarino P, Boutroy JL, Gueant JL (2002). Hyperhomocysteinemia is related to a decreased blood level of vitamin B12 in the second and third trimester of normal pregnancy. Clin Chem Lab Med.

[35] Delabar U, Kloor D, Luippold G, Muhlbauer B (1999). Simultaneous determination of adenosine, S-adenosylhomocysteine and S-adenosylmethionine in biological samples using solid-phase extraction and high-performance liquid chromatography. J Chromatogr B Biomed Sci Appl.

[36] Wang BX, Yin BL, He B, Chen C, Zhao M, Zhang WX, Xia ZK, Pan YZ (2012). Overexpression of DNA damage-induced 45 α gene contributes to esophageal squamous cell cancer by promoter hypomethylation. J Exp Clin Cancer Res.

[37] Wallin J, Wilting J, Koseki H, Fritsch R, Christ B, Balling R (1994). The role of Pax-1 in axial skeleton development. Development.

[38] Eves EM, Tucker MS, Roback JD, Downen M, Rosne MR, Wainer BH (1992). Immortal rat hippocampal cell lines exhibit neuronal and glial lineages and neurotrophin gene expression. Proc Natl Acad Sci U S A.

[39] Akchiche N, Bossenmeyer-Pourié C, Pourié G, Koziel V, Nédélec E, Guéant JL, Daval JL (2010). Differentiation and neural integration of hippocampal neuronal progenitors: signaling pathways sequentially involved. Hippocampus.

[40] Kloosterman WP, Wienholds E, de Bruijn E, Kauppinen S, Plasterk RH (2006). In situ detection of miRNAs in animal embryos using LNA-modified oligonucleotide probes. Nat Methods.

[41] Tonkiss J, Harrison RH, Galler JR (1996). Differential effects of prenatal protein malnutrition and prenatal cocaine on a test of homing behavior in rat pups. Physiol Behav.

[42] Slamberová R, Pometlová M, Charousová P (2006). Postnatal development of rat pups is altered by prenatal methamphetamine exposure. Prog Neuro-Psychopharmacol Biol Psychiatry.

[43] El Hajj Chehadeh S, Pourié G, Martin N, Alberto JM, Daval JL, Guéant JL, Leininger-Muller B (2014). Gestational methyl donor deficiency alters key proteins involved in neurosteroidogenesis in the olfactory bulbs of newborn female rats and is associated with impaired olfactory performance. Br J Nutr.

[44] Bâ A (2013). Perinatal thiamine deficiency-induced spontaneous abortion and pup-killing responses in rat dams. Nutr Neurosci.

[45] Ecsedi M, Grosshans H (2013). LIN-41/TRIM71: emancipation of a miRNA target. Genes Dev.

[46] Ishibashi M (2004). Molecular mechanisms for morphogenesis of the central nervous system in mammals. Anat Sci Int.

[47] Desai A, Sequeira JM, Quadros EV (2016). The metabolic basis for developmental disorders due to defective folate transport. Biochimie.

[48] Lambrot R, Xu C, Saint-Phar S, Chountalos G, Cohen T, Paquet M, Suderman M, Hallett M (2013). Low paternal dietary folate alters the mouse sperm epigenome and is associated with negative pregnancy outcomes. Nat Commun.

[49] Beaudin AE, Stover PJ (2007). Folate-mediated one-carbon metabolism and neural tube defects: balancing genome synthesis and gene expression. Birth Defects Res C Embryo Today.

[50] Greene ND, Dunlevy LE, Copp AJ (2003). Homocysteine is embryotoxic but does not cause neural tube defects in mouse embryos. Anat Embryol (Berl).

[51] Copp AJ, Greene ND (2013). Neural tube defects—disorders of neurulation and related embryonic processes. Wiley Interdiscip Rev Dev Biol.

[52] Meza-Sosa K, Valle-García D, Pedraza-Alva G, Pérez-Martínez L (2012). Role of microRNAs in central nervous system development and pathology. J Neurosci Res.

[53] Boland MJ, Nazor KL, Loring JF (2014). Epigenetic regulation of pluripotency and differentiation. Circ Res.

[54] Meza-Sosa KF, Pedraza-Alva G, Pérez-Martínez L (2014). microRNAs: key triggers of neuronal cell fate. Front Cell Neurosci.

[55] Büssing I, Slack FJ, Grosshans H (2008). Let-7 microRNAs in development, stem cells and cancer. Trends Mol Med.

[56] Cimadamore F, Amador-Arjona A, Chen C, Huang CT, Terskikh AV (2013). SOX2-LIN28/let-7 pathway regulates proliferation and neurogenesis in neural precursors. Proc Natl Acad Sci U S A.

[57] Hartl M, Grunwald Kadow IC (2013). New roles for “old” microRNAs in nervous system function and disease. Front Mol Neurosci.

[58] Lin YC, Hsieh LC, Kuo MW, Yu J, Kuo HH, Lo WL, Lin RJ, Yu AL (2007). Human TRIM71 and its nematode homologue are targets of let-7 microRNA and its zebrafish orthologue is essential for development. Mol Biol Evol.

[59] Rybak A, Fuchs H, Hadian K, Smirnova L, Wulczyn EA, Michel G, Nitsch R, Krappmann D (2009). The let-7 target gene mouse lin-41 is a stem cell specific E3 ubiquitin ligase for the miRNA pathway protein Ago2. Nat Cell Biol.

[60] Chang HM, Martinez NJ, Thornton JE, Hagan JP, Nguyen KD, Gregory RI (2012). Trim71 cooperates with microRNAs to repress Cdkn1a expression and promote embryonic stem cell proliferation. Nat Commun.

[61] Thomson JM, Parker J, Perou CM, Hammond SM (2004). A custom microarray platform for analysis of microRNA gene expression. Nat Methods.

[62] Chen F, Hu SJ (2012). Effect of microRNA-34a in cell cycle, differentiation, and apoptosis: a review. J Biochem Mol Toxicol.

[63] Machka C, Kersten M, Zobawa M, Harder A, Horsch M, Halder T, Lottspeich F, Hrabé de Angelis M (2005). Identification of Dll1 (Delta1) target genes during mouse embryogenesis using differential expression profiling. Gene Expr Patterns.

[64] Kageyama R, Ohtsuka T, Hatakeyama J, Ohsawa R (2005). Roles of bHLH genes in neural stem cell differentiation. Exp Cell Res.

[65] Hatakeyama J, Bessho Y, Katoh K, Ookawara S, Fujioka M, Guillemot F, Kageyama R (2004). Hes genes regulate size, shape, and histogenesis of the nervous system by control of the timing of neural stem cell differentiation. Development.

[66] Copp AJ, Greene ND, Murdoch JN (2003). The genetic basis of mammalian neurulation. Nat Rev Genet.

[67] Agostini M, Tucci P, Steinert JR, Shalom-Feuerstein R, Rouleau M, Aberdam D, Forsythe ID, Young KW (2011). microRNA-34a regulates neurite outgrowth, spinal morphology, and function. Proc Natl Acad Sci USA.

[68] Bossenmeyer-Pourié C, Blaise S, Pourié G, Tomasetto C, Audonnet S, Ortiou S, Koziel V, Rio MC (2010). Methyl donor deficiency affects fetal programming of gastric ghrelin cell organization and function in the rat. Am J Pathol.

[69] Craciunescu CN, Brown EC, Mar MH, Albright CD, Nadeau MR, Zeisel SH (2004). Folic acid deficiency during late gestation decreases progenitor cell proliferation and increases apoptosis in fetal mouse brain. J Nutr.

[70] Craciunescu CN, Johnson AR, Zeisel SH (2010). Dietary choline reverses some, but not all, effects of folate deficiency on neurogenesis and apoptosis in fetal mouse brain. J Nutr.

[71] Raznahan A, Greenstein D, Lee NR, Clasen LS, Giedd JN (2012). Prenatal growth in humans and postnatal brain maturation into late adolescence. Proc Natl Acad Sci USA.

[72] Botto LD, Yang Q (2000). 5,10-methylenetetrahydrofolate reductase gene variants and congenital anomalies: a HuGE review. Am J Epidemiol.

[73] Yan L, Zhao L, Long Y, Zou P, Ji G, Gu A, Zhao P (2012). Association of the maternal MTHFR C677T polymorphism with susceptibility to neural tube defects in offsprings: evidence from 25 case-control studies. PLoS One.

[74] Blaise SA, Nédélec E, Alberto JM, Schroeder H, Audonnet S, Bossenmeyer-Pourié C, Guéant JL, Daval JL (2009). Short hypoxia could attenuate the adverse effects of hyperhomocysteinemia on the developing rat brain by inducing neurogenesis. Exp Neurol.

[75] Zhang X, Liu H, Cong G, Tian Z, Ren D, Wilson JX, Huang G (2008). Effects of folate on notch signaling and cell proliferation in neural stem cells of neonatal rats in vitro. J Nutr Sci Vitaminol (Tokyo).

[76] Liu H, Huang GW, Zhang XM, Ren DL, Wilson XJ (2010). Folic acid supplementation stimulates notch signaling and cell proliferation in embryonic neural stem cells. J Clin Biochem Nutr.

[77] Ichi S, Costa FF, Bischof JM, Nakazaki H, Shen YW, Boshnjaku V, Sharma S, Mania-Farnell B (2010). Folic acid remodels chromatin on Hes1 and Neurog2 promoters during caudal neural tube development. J Biol Chem.

[78] Li Y, Saldanha SN, Tollefsbol TO (2014). Impact of epigenetic dietary compounds on transgenerational prevention of human diseases. AAPS J.

