# Supplementary material for: Late Maternal Folate Supplementation Rescues from Methyl Donor Deficiency-Associated Brain Defects by Restoring Let-7 and miR-34 Pathways
Source: Mol Neurobiol. 2016 Aug 17;54(7):5017–33. doi: 10.1007/s12035-016-0035-8 (PMC5533871; doi:10.1007/s12035-016-0035-8)
Supplement: Supplementary file 1 — Effects of methyl donor deficiency and folic acid supplementation on the expression of let-7a as depicted by in situ hybridization in the hippocampus, cerebellum and cerebral cortex from E20 fetuses (PDF 230 kb) [file 12035_2016_35_MOESM1_ESM.pdf]

miR-34a

Cell migration, axon guidance, cytoskeleton			Down-regulation compared to control	
Symbol	Name	Function	MDD	MDD-B9
FoxP1	Forkhead box P1	Neuron migration, axon guidance, embryonic development	-6.05	-2.08**
Nav3	Neuron navigator 3	Neuron navigator family, axon guidance	-6.19	-3.26*
Nrn1	Neuritin 1	Promote neurites outgrowth	-4.94	-3.05*
Cd44	Cd44 molecule	Adhesion molecule, cell migration, cartilage development, Wnt signaling	-2.45	-2.36
Myh9	Myosin, heavy chain 9, non-muscle	Cell motility	-4.35	-3.04
Coroc1c	Coronin, actin binding protein 1C	Cell motility	-5.52	-2.78**
Arpp19	CAMP-regulated phosphoprotein 19	NGF signaling, post-transcritional control of neuronal genes expression.	-5.16	-2.76**
Ctnnd2	Catenin (cadherin-associated protein), delta 2	Cellular junction, post-synaptic density, brain development	-4.91	-2.73*

Cell cycle			Down-regulation compared to control	
Ccne2	Cyclin E2	G1/S transition, regulates Cdk2	-4.69	-2.74*
Cdc25a	Cell division cycle 25 homolog A	G1/S transition, DNA damage	-5.29	-2.88**
Cdk4	Cyclin-dependent kinase 4	G1/S and G2/M transition	-4.73	-2.42*
Cdk6	Cyclin-dependent kinase 6	G/1S transition, Notch signaling	-4.17	-2.07**
E2f3	E2F transcription factor 3	Bind to pRB to regulate the expression of genes involved in the cell cycle	-5.22	-2.74*
Myc	Myelocytomatosis oncogene	p53-dependent G2 arrest, Notch signaling, Wnt signaling	-6.69	-2.54**

Vesicular trafficking, synapse			Down-regulation compared to control	
Flot2	Flotillin 2	Neuronal signaling, caveloae-associated membrane protein	-5.32	-2.64**
Syt1	Synaptotagmin I	Neurotransmitter exocytosis and vesicle trafficking	-5.05	-2.99*
Syn2	Synapsin II	Synaptic vesicle trafficking	-5.73	-2.89**
Vat1	Vesicle amine transport protein 1 homolog	Cholinergic vesicle trafficking	-4.90	-2.83*
Neto1	Neuropilin (NRP) and tolloid (TLL)-like 1	Synaptic plasticity	-6.19	-2.28**
Sar1a	SAR1 homolog A	Vesicle mediated transport	-4.62	-2.53*
Trim9	Tripartite motif-containing 9	Synaptic vesicle exocytosis	-5.45	-2.88*
Snx15	Sorting nexin 15	Endosomal trafficking	-4.96	2.46**
