# Supplementary material for: Late Maternal Folate Supplementation Rescues from Methyl Donor Deficiency-Associated Brain Defects by Restoring Let-7 and miR-34 Pathways
Source: Mol Neurobiol. 2016 Aug 17;54(7):5017–33. doi: 10.1007/s12035-016-0035-8 (PMC5533871; doi:10.1007/s12035-016-0035-8)
Supplement: Supplementary file 2 — Effects of methyl donor deficiency and folic acid supplementation on the expression of miR-34a as depicted by in situ hybridization in the hippocampus, cerebellum and cerebral cortex from E20 fetuses (PDF 201 kb) [file 12035_2016_35_MOESM2_ESM.pdf]

Let-7a

Cell migration, axon guidance, cytoskeleton			Down-regulation compared to control	
**Symbol**	**Name**	**Function**	**MDD**	**MDD-B9**
Itgb3	Integrin, beta 3	Cell motility	-4.42	-2.73 *
Lrig2	Leucine-rich repeats and immunoglobulin-like domains 2	Innervation, neurite outgrowth, synapses formation	-5.92	-2.89*
Nf2	Neurofibromin 2 (merlin)	Cytoskeleton organization, embryonic development	-5.09	-2.28**
Plxnc1	Plxnc1	Cell motility, axon guidance	-6.82	-3.07**
Coll3a1	Collagen, type III, alpha 1	Axon guidance, cerebral cortex development	-3.94	-2.70
**Cell cycle**			**Down-regulation compared to control**	
Ccna2	Cyclin A2	G1/S and G/2M transition	-4.69	-1.82**
Ccnd1	Cyclin D1	G/1S transition, Notch signaling	-5.05	-3.00*
Ccnd2	Cyclin D2	G1/S transition	-4.42	-1.63**
Cdc25a	Cell division cycle 25 homolog A	G1/S transition, DNA damage	-5.48	-2.56*
Cdc34	Cell division cycle 34 homolog	ubiquitin-mediated degradation of cell cycle G1 regulators	-5.39	-2.53*
Cdk6	Cyclin-dependent kinase 6	G/1S transition, Notch signaling	-4.43	-1.97**
E2f1	E2F transcription factor 1	G/1S transition, Notch signaling, DNA damage	-3.52	-2.19
Uhrf2	Ubiquitin-like with PHD and ring finger domains 2	G1 arrest	-4.99	-2.28**
Myc	Myelocytomatosis oncogene	p53-dependent G2 arrest, Notch signaling, Wnt signaling	-6.47	-2.63**
Ppp2r2a	Protein phosphatase 2, regulatory subunit B, alpha isoform	G2/M transition	-5.59	-2.39*
Cntrl	Centrosomal protein 110	G2/M transition	-6.10	-2.32**
Cep135	Centrosomal protein 135	G2/M transition, implicated in microcephaly	-6.05	-2.27**
**RNA trafficking**			**Down-regulation compared to control**	
Dicer	Dicer 1, ribonuclease type III	miRNA processing, cerebral cortex developement	-5.79	-2.16**
IGFBP1	Insulin-like growth factor 2 mRNA binding protein 1	mRNA stability	-3.43	-1.81*
Raver2	Ribonucleoprotein, PTB-binding 2	Poly(A) RNA-binding protein	-6.30	-3.02**
Lin28a	Lin-28 homolog	Pre-miRNA processing	-11.70	-11.71
IGFBP3	Insulin-like growth factor 2 mRNA binding protein 3	RNA binding, regulation of translation during late development	-4.71	-2.35*
Ago4	Eukaryotic translation initiation factor 2C, 4	SiRNA mediated gene silencing, Stem cell maintenance	-5.87	-2.71**
Ddx19b	DEAD (Asp-Glu-Ala-As) box polypeptide 19B	RNA helicase, mRNA export from the nucleus	-5.14	-2.21**
