# Supplementary figures and images for: Late Maternal Folate Supplementation Rescues from Methyl Donor Deficiency-Associated Brain Defects by Restoring Let-7 and miR-34 Pathways
Source: Mol Neurobiol. 2016 Aug 17;54(7):5017–33. doi: 10.1007/s12035-016-0035-8 (PMC5533871; doi:10.1007/s12035-016-0035-8)

a. **Let-7/DAPI - Hippocampus**

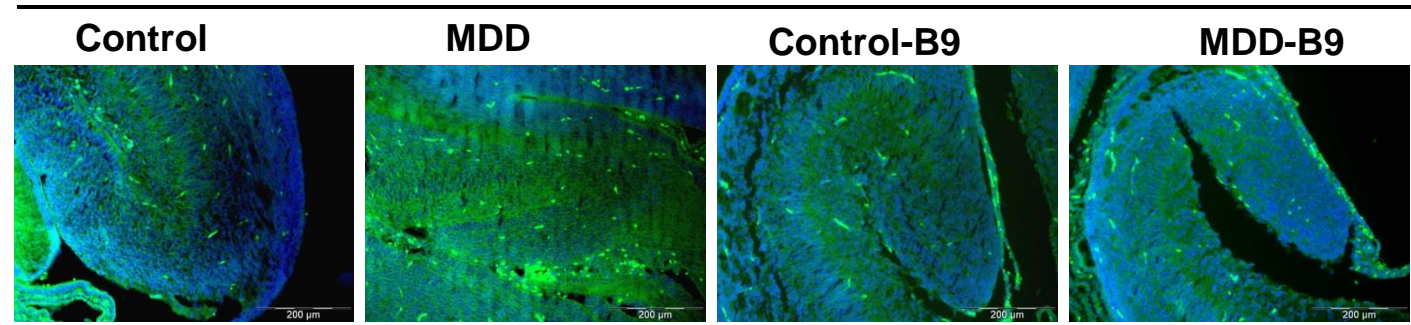

b. **Let-7/DAPI - Cerebellum**

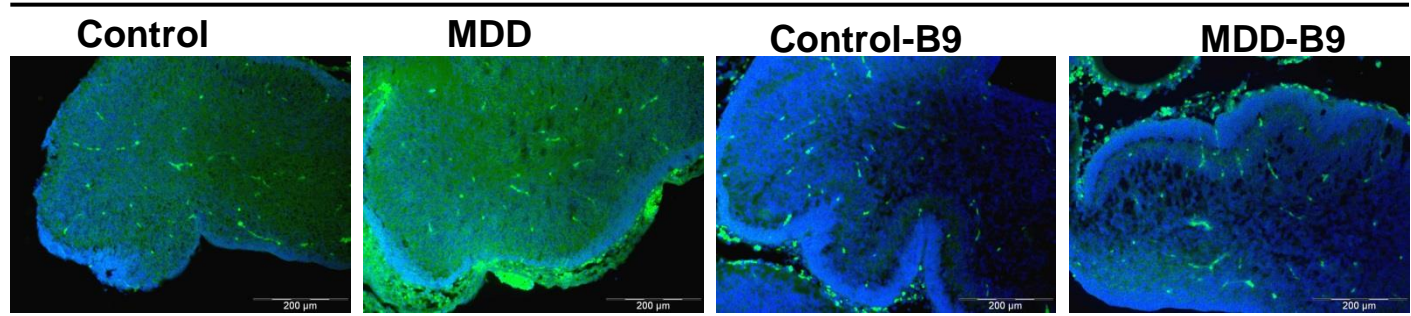

c. **Let-7/DAPI - Cortex**

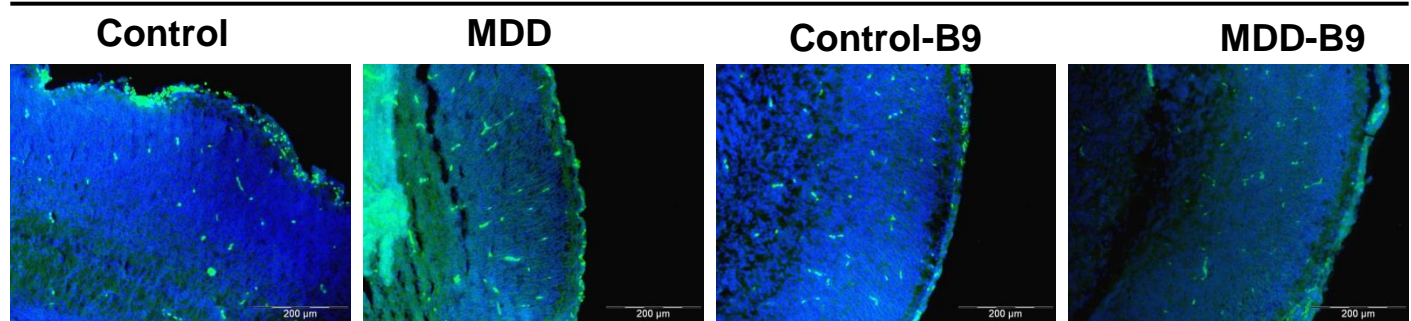

Supplement: Supplementary file 3 — Consequences of miR-34a silencing on the morphology of control and folate-deficient (MDD) H19–7 cells at 13 h after induction of their differentiation (Si- = non-targeting siRNA, Si-miR-34a = miR-34a targeted siRNA). Cells are colabeled with antibodies against actin (green) and NF68 (red) and their nuclei counterstained by Dapi (blue) (PDF 150 kb) [file 12035_2016_35_MOESM3_ESM.pdf]

a. **miR34/DAPI - Hippocampus**

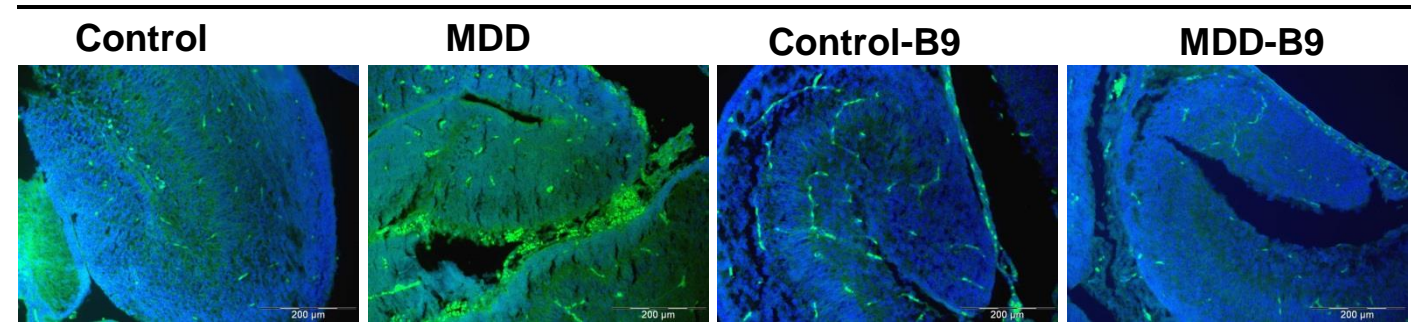

b. **miR34/DAPI - Cerebellum**

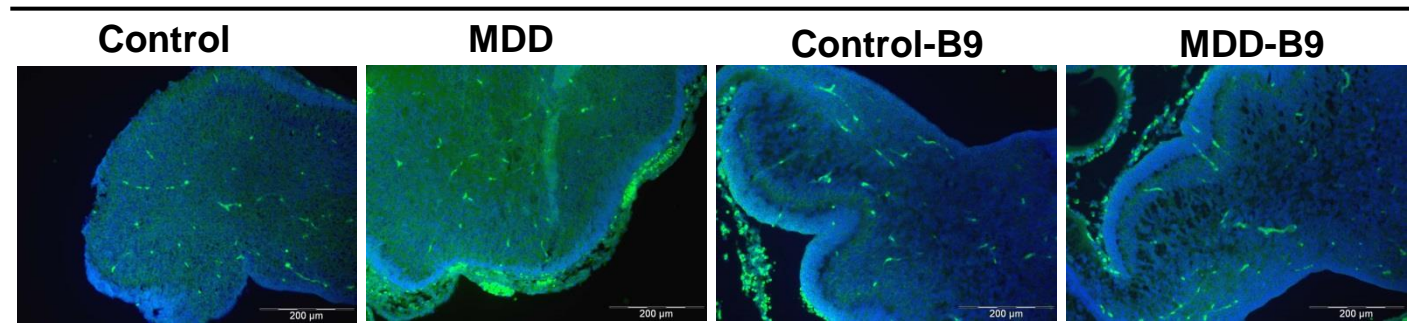

c. **mir34/DAPI - Cortex**

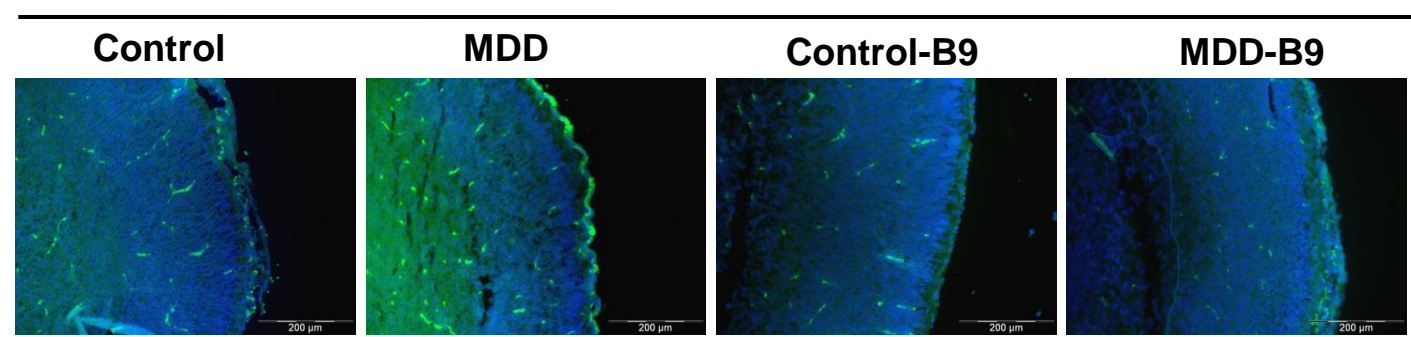

Supplement: Supplementary file 4 — Downregulation of let-7a gene targets, according to their known functions, in methyl donor deficient (MDD) and supplemented deficient (MDD-B9) brain fetuses. Statistically significant differences between MMD and MDD-B9: *P < 0.05, **P < 0.01 (PDF 176 kb) [file 12035_2016_35_MOESM4_ESM.pdf]

Actin / NF68 / Dapi

Control Si-

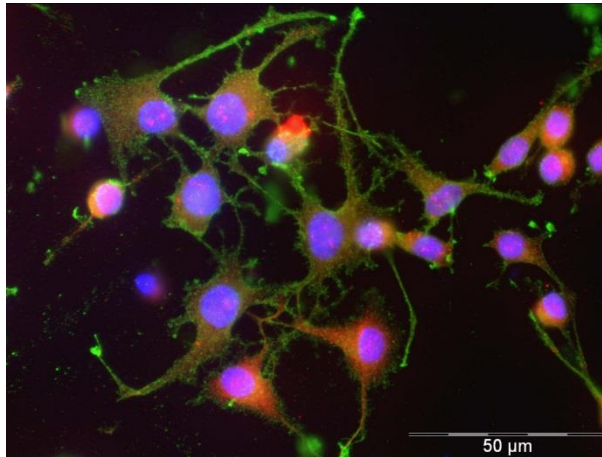

Control Si-miR34a

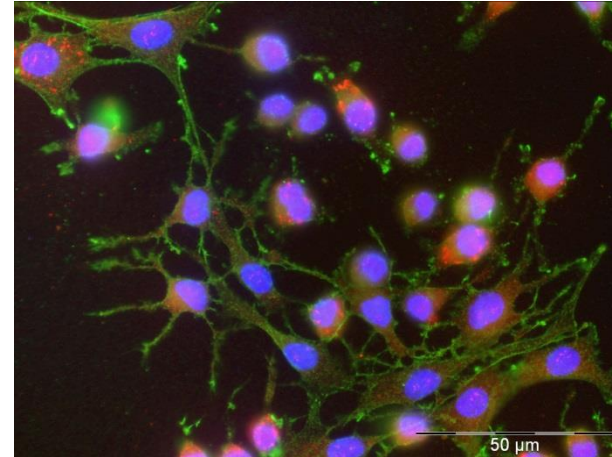

MDD Si-

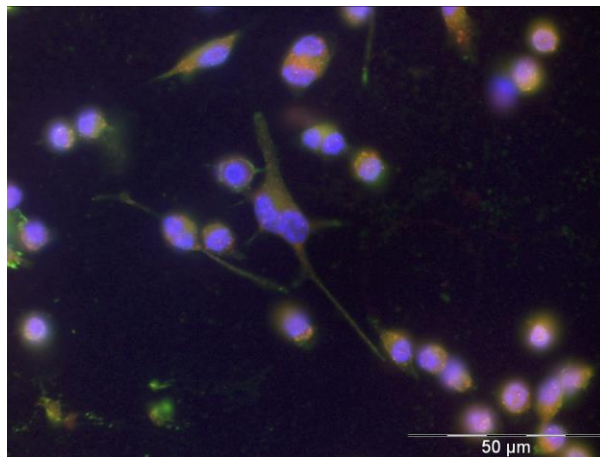

MDD Si-miR34a

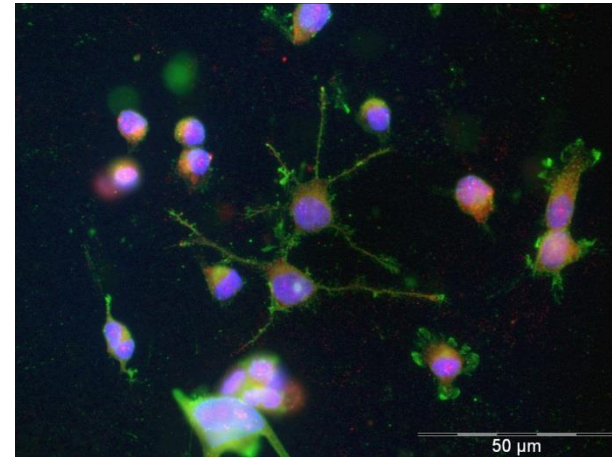

Supplement: Supplementary file 5 — Downregulation of miR-34a gene targets, according to their known functions, in methyl donor deficient (MDD) and supplemented deficient (MDD-B9) brain fetuses. Statistically significant differences between MMD and MDD-B9: *P < 0.05, **P < 0.01 (PDF 175 kb) [file 12035_2016_35_MOESM5_ESM.pdf]
